# An LLM-assisted real-time simulation framework for demand-responsive transport operations

**DOI:** 10.1371/journal.pone.0360420

**Published:** 2026-10-27

**Authors:** Jun-Woo Kim, Min-Ho Choi, Sun-Woo Lee, Kyung-Min Seo

**Affiliations:** 1 Department of Industrial and Management Engineering, Hanyang University, Seongdong-gu, Seoul, Republic of Korea; 2 Department of Industrial and Management Engineering, Hanyang University ERICA, Ansan, Gyeonggi-do, Republic of Korea; Alma Mater Studiorum Universita di Bologna: Universita degli Studi di Bologna, ITALY

## Abstract

Demand-responsive transport (DRT) is a dynamic service whose performance depends not only on dispatching and routing algorithms but also on how externally submitted demand is captured, interpreted, and incorporated into operations. Although prior studies have largely emphasized algorithm-level optimization using pre-defined demand scenarios, integrated validation of externally submitted demand, user- and operator-facing interfaces, and operational analytics remains limited. This study proposes an LLM-assisted real-time DRT operation framework comprising Service User, System Manager, and Real-time DRT Simulator layers. The Service User layer uses function calling to transform unstructured trip requests into structured dispatch parameters. The System Manager combines function-calling-based quantitative analysis with retrieval-augmented generation to support natural-language access to operational data and domain knowledge. Based on Discrete Event System Specification, the simulator processes externally submitted and simulation-generated demands within a unified event structure under common dispatching and routing logic. Three experiments were conducted. Experiments 1 and 2 functionally evaluated the rider request-processing interface and the function-calling-based quantitative-query path of the System Manager, yielding average response latencies of 1.22 and 2.21 s, respectively. Experiment 3 independently evaluated the simulator through a system-level stress test using context-based synchronized demand. Across all scenarios, synchronized demand increased waiting times for the target passenger group. Although this group represented approximately 20% of successfully served passengers, it accounted for 26.5–28.5% of total waiting time. The effect appeared largely localized and was partially obscured in aggregate metrics because background-passenger performance was stable. These results demonstrate the functional feasibility of the LLM-assisted interfaces under the prototype conditions and show that synchronized-demand stress testing can reveal localized delay burdens that aggregate evaluations may overlook.

## 1. Introduction

Demand Responsive Transport (DRT) is a transportation service in which vehicle dispatching and routing are adjusted dynamically in response to user ride requests. Unlike traditional bus systems, which operate on fixed routes and schedules, DRT determines vehicle stop sequences and routes by aggregating requests from multiple users once their origins and destinations are submitted. Owing to these characteristics, DRT has attracted attention as an alternative to fixed-route services in areas with low population density or temporally and spatially imbalanced demand [1–6]. In particular, demand-adaptive operating strategies are needed in environments such as transport-disadvantaged areas and university campuses, where demand fluctuates substantially by time of day, and fixed-route services often suffer from detours and underutilization [6–13].

The operational performance of DRT is determined by the interaction of multiple factors, including the passenger distribution, fleet size, dispatching policies, and vehicle routing. In academic literature, DRT has traditionally been formulated as a Dial-a-Ride Problem (DARP), and a wide range of mathematical optimization and heuristic algorithms have been proposed [14–17]. Real-time matching algorithms for large-scale on-demand shared mobility systems have further advanced the computational foundation of this field [18–21]. Although these studies have made important contributions to improving the efficiency of vehicle dispatching and routing, they tend to focus primarily on algorithm-level performance comparisons and the analysis of design variables.

However, DRT is not merely an optimization problem; it is a real-time service system in which the request intake, dispatching, operation, and policy adjustment interact continuously. In real-world service settings, passenger requests arrive continuously and operators must interpret and respond to changing operational conditions. Nevertheless, much of the existing literature has focused on evaluating algorithm performance or policy effects based on pre-defined demand scenarios, namely, virtual demand. Although this approach is useful for verifying dispatching and routing logic under controlled conditions, it does not adequately capture the stochastic nature of externally submitted demand or user- and operator-facing interactions that arise in real-world operations [22–25].

Operations in real-world environments introduce complexities beyond demand generation. First, the spatial and temporal distributions of the actual demand are highly stochastic and driven by irregular user behavior rather than by pre-defined probabilistic models. Second, capturing and processing such demands inevitably involves human–system interactions, in which users may specify origins or destinations using landmarks, informal references, or natural language expressions containing typographical errors [26]. These factors directly affect operational outcomes, including the input quality for dispatching, route planning, service rejection rates, and passenger waiting times, and are difficult to evaluate fully through offline simulations alone [14,27].

Simultaneously, the applicability of Large Language Models (LLMs) to transportation system analysis and decision support has recently expanded [28–31]. In transportation and Intelligent Transportation Systems (ITS), LLMs are increasingly being explored for natural language answering, policy analysis assistance, and automation of traffic data interpretation [32–34]. Their potential has also been suggested for public transport data analysis and customer response automation [32]. This implies that LLMs can help reduce the burden of interpreting complex transportation data and support operational decision-making [30,31]. Particularly in services such as DRT, where user requests and operational judgments occur frequently, an interface layer that converts natural language input into structured operational information and enables operators to query and interpret the required performance indicators in real-time is important.

Nevertheless, studies integrating LLMs into request handling and operational analysis within DRT systems and comprehensively validating them in real-time simulation environments remain limited. In particular, previous studies have separately addressed LLM-based transportation data retrieval, LLM-agent-based mobility modeling, and RAG-based travel behavior prediction; however, these functions have rarely been integrated into the actual operational workflow of DRT services. Specifically, studies combining natural language request intake, structured dispatch parameter generation, operator-side KPI analysis, and real-time simulation-based validation within a unified DRT event-processing framework remain particularly limited. To address this gap, this study proposes a DRT operation framework that integrates LLM-based request handling and operational analysis within a real-time simulation environment. The proposed framework consists of three interconnected layers: the Service User, System Manager, and Real-time DRT Simulator, all of which are linked through a shared database and an event-processing structure. In the Service User layer, the natural language requests of the user are structured through function calling and transformed into inputs that can be processed by a dispatching module [31,35]. In the System Manager layer, function-calling-based quantitative analysis is combined with retrieval-augmented generation (RAG)-based question answering [36,37] to support operators in querying and interpreting key performance indicators (KPIs). The Real-time DRT Simulator processes externally submitted and simulation-generated demands within a unified event structure and verifies the dispatching and routing logic under consistent service rules in a Discrete Event System Specification (DEVS)-based execution environment [38–40].

To evaluate the proposed framework, a three-stage validation strategy was designed. Experiment 1 evaluated whether an LLM-based chatbot could rapidly and accurately convert unstructured natural language requests into the structured dispatching parameters required by the system. Experiment 2 examined whether the LLM-based manager query interface could interpret natural-language queries from operators and accurately perform pre-defined KPI computations and status monitoring with low response latency. Finally, Experiment 3 conducted a stress test in a real-time simulation environment by injecting context-based synchronized demand that mimicked actual demand concentration. This experiment enabled an in-depth analysis of context-dependent operational vulnerabilities and delay propagation patterns in DRT systems, which are difficult to capture through conventional virtual demand-based evaluation using both macroscopic and microscopic indicators.

The main contributions of this study are as follows. First, a DEVS-based real-time DRT simulation framework is proposed that processes externally submitted and simulation-generated demands within a unified event structure [39, 40]. This allows externally submitted and simulation-generated demands to be processed under the same dispatching and routing logic and service rules, thereby enabling consistent validation of operating strategies regardless of the source of demand. Second, a dual LLM interface is designed and implemented to support both user-side natural language request handling and operator-side KPI querying and interpretation. On the user side, function-calling-based request structuring is applied [31,35], whereas on the operator side, function-calling-based quantitative analysis is combined with RAG-based question answering [36,37], thereby realizing a natural language interaction structure that addresses the needs of different operational actors. Third, the study evaluates the proposed framework through a layered validation strategy. Experiment 1 evaluates the accuracy and responsiveness of the LLM-based user interface for structured request handling, whereas Experiment 2 evaluates the function-calling-based quantitative-query path of the System Manager for KPI computation and vehicle-status monitoring. The RAG-based knowledge-retrieval path is implemented but is not separately validated in this study. Experiment 3 evaluates the Real-time DRT Simulator through a context-based synchronized-demand stress test and demonstrates that context-based synchronized demand can reveal localized operational vulnerabilities that are difficult to observe through conventional aggregate-level virtual demand evaluations.

The remainder of this paper is organized as follows. Section 2 reviews the related studies and clarifies the distinctions of the present research. Section 3 describes the system architecture and the implementation of the proposed framework. Section 4 presents the experimental design and performance evaluation results. Finally, Section 5 concludes the paper and discusses directions for future research.

## 2. Related work

Existing DRT studies have primarily relied on optimization- and simulation-based analyses. David et al. [7] investigated the DRT concept and examined its potential to improve service quality using Simulation of Urban Mobility (SUMO)-based simulations. They conducted sensitivity analyses focusing on vehicle dispatching and route optimization and used simulations to compare service levels. However, the demand was generated from scenario inputs based on precalculated boarding and alighting parameters, and neither real-time call flows nor user interactions were considered. Kagho et al. [5] analyzed the effects of introducing DRT in a specific region using a MATSim-based agent simulation [41]. They modeled demand at the agent level and reproduced regional traffic patterns to evaluate policy effects. However, the demand was generated based on pre-defined fixed rates and no framework was presented to incorporate dynamic operational factors, such as real-time call arrivals or operator-side monitoring and analysis. Kim et al. [8] analyzed the benefits of regional DRTs using actual smart card data. They provided empirical evidence by extracting route-level demand volumes and patterns from historical records and comparing their performances. However, they adopted a retrospective analytical approach and did not address the call handling or operational decision-making processes in real-time operating environments. Cho et al. [9] proposed a rapid DRT service strategy using a DEVS-based discrete event simulation framework [38,39]. They presented a system-level analytical structure through an experimental comparison of various operating rules, including express insertion strategies. However, the demand was still defined based on virtual scenarios and was not extended to an integrated real-time simulation framework that combines externally submitted user requests with operator-side operational analysis functions.

DRT and flexible transit services have also been studied from the perspectives of agent-based simulation, service design, and online operations under uncertain demand. Ronald et al. [23] reviewed agent-based approaches for simulating demand-responsive transport and emphasized the importance of modeling interactions among passengers, operators, and vehicles. Zhao et al. [42] investigated the joint optimization of regular public transport and demand-responsive transport services, demonstrating that fixed-route and demand-responsive services can be designed to complement each other. Lee et al. [43] jointly optimized zoning, scheduling, and fares for flexible bus services under spatial and temporal demand uncertainty. Li et al. [44] proposed a framework integrating offline planning and online operations for zonal-based flexible transit services under uncertain demand and dynamic cancellations. These studies demonstrate that the performance of DRT and flexible transit systems is strongly influenced by demand uncertainty, service-area design, vehicle operations, and real-time operational decision-making. In parallel with research on DRT operations, recent studies have explored the application of LLMs in transportation systems. Nie et al. [45] presented a survey and roadmap for LLMs in transportation and categorized their roles into information processing, knowledge encoding, component generation, and decision facilitation. Liu et al. [46] proposed an LLM-agent-based transportation modeling framework to improve behavioral realism and interaction modeling in transportation simulation. Devunuri et al. [47] evaluated LLMs on GTFS semantic understanding and retrieval tasks, demonstrating that natural language interfaces can support access to structured public transport data. Xu and Jiao [48] examined RAG strategies for LLM-based travel mode choice prediction and emphasized the importance of grounding LLM responses in empirical transportation data. These studies suggest that LLMs can enhance transportation systems by converting natural language into structured queries, supporting data retrieval, and improving user access to complex operational information.

Although these studies have made meaningful contributions to DRT optimization, flexible transit design, demand uncertainty modeling, and LLM-based transportation analysis, several limitations remain from the perspective of platform-level operational validation. First, most DRT studies generate demand from pre-defined scenarios, historical records, or assumptions embedded within the model, thereby failing to sufficiently reflect real-time request intake and user- and operator-facing interactions. Second, transportation studies involving LLMs have focused primarily on data retrieval, travel behavior modeling, or conceptual modeling frameworks, without integrating LLM interfaces into the operational workflow of DRT services. Third, few studies have connected user-side natural language request processing, operator-side KPI analysis, and real-time dispatching simulation within a unified event-processing structure. Consequently, previous studies have not sufficiently presented an integrated operational validation structure that integrates request intake, dispatching, operation, and analysis. In contrast, this study proposes an LLM-assisted real-time DRT operation framework that supports natural language interactions for both service users and system operators while processing externally submitted and simulation-generated demands under the same dispatching and routing logic. The following section describes in detail the overall architecture and constituent modules of the proposed framework. Table 1 summarizes the research scope, methods, analysis types, and demand-management characteristics of the reviewed studies and compares them with the present study.

**Table 1:** Summary of related studies on DRT operations and LLM-assisted transportation interfaces.

Study	Research Content	Proposed Method	Analysis Type	Demand Management & Real-time Operation
David et al.[7]	Vehicle dispatch and route optimization	SUMO-based sensitivity analysis	General statistical analysis	Potential passenger demand was generated from observed boarding and alighting factors, and the analysis was conducted based on virtual demand scenarios.
Kagho et al.[5]	Introduction of DRT in regions with high private car dependency	Agent-based demand modeling [41]	General statistical analysis	Demand in specific regions was modeled as fixed agent-based demand and generated through sampling; however, the real-time call flow was not reflected.
Kim et al. [8]	Performance analysis of DRT systems	Region-based demand separation and smart card-based demand generation	General statistical analysis	Route-level demand scale and patterns were extracted from historical records; however, the real-time call flow was not reflected.
Cho et al. [9]	Proposal of a rapid DRT service	Express insertion optimization (DEVS) [38,39]	General statistical analysis	Demand was estimated by applying taxi occupancy rates to smart card-based stop demand; however, validation with externally submitted demand was not included.
Ronald et al. [23]	Review of DRT simulation studies	Agent-based simulation review	Literature review	Emphasized the importance of modeling interactions among passengers, vehicles, and operators, but did not propose a real-time operational platform with natural-language interfaces.
Lee et al. [43]	Flexible bus service design under spatial and temporal demand uncertainty	Joint optimization of zoning, scheduling, and pricing	Optimization-based analysis	Considered spatial and temporal demand uncertainty but did not integrate real-time user request intake or operator-side LLM analytics.
Li et al. [44]	Offline planning and online operation of zonal-based flexible transit	Planning and online routing under uncertain demand and cancellations	Optimization and online operation	Considered online operation under real-time orders and cancellations but did not address LLM-based request handling or integrated user- and operator-facing simulation interfaces.
Devunuri et al. [47]	Natural-language access to structured transit data	LLM benchmarking on GTFS semantics and retrieval	LLM benchmark	Demonstrated LLM-based retrieval of structured transit data but did not connect the interface to real-time DRT dispatching and simulation.
This Study	Layered functional and system-level validation of DRT operations integrating externally submitted demand, simulation-generated demand, and user- and operator-facing interactions	LLM-assisted real-time simulation framework with a DEVS-based real-time simulator and dual natural language interfaces	Functional verification and system-level stress testing	Externally submitted and simulation-generated demands are processed in a unified event structure under shared dispatching and routing logic, enabling real-time user interaction, operator monitoring, and KPI-based analysis.

## 3. Methods

### 3.1 Proposed framework

This section describes the structural design of the proposed LLM-assisted real-time DRT operation framework. The proposed framework is designed with three primary objectives: integrated processing of externally submitted and simulation-generated demands, support for real-time user- and operator-facing interactions, and provision of dual natural language interfaces for both operators and users. To achieve these objectives, the framework adopts three functionally separate layers (Service User, System Manager, and Real-time DRT Simulator) that are interconnected through a centralized database architecture. First, the overall system architecture is presented, followed by a sequential description of the internal components and processing pipeline of each layer.

#### 3.1.1 Overall system architecture.

Fig 1 illustrates the overall system architecture of the proposed LLM-assisted real-time DRT operation framework. As previously mentioned, the architecture operates across three core layers. The Service User layer collects ride requests from users, whereas the System Manager layer provides operators with real-time monitoring and analysis functions. The Real-time DRT Simulator serves as the core execution layer, performing vehicle dispatching and routing while jointly processing both externally submitted and simulation-generated demands. The key design principle of the system is to process externally submitted and simulation-generated demands within a unified event structure, ensuring that both are handled under the same vehicle dispatching and routing logic as well as identical service rules. To this end, user requests submitted in the Service User layer are delivered to the Real-time DRT Simulator, which returns the corresponding dispatching and operational results to the user. Concurrently, the data generated by the simulator is stored in a centralized database. Both the Service User and System Manager layers retrieve relevant information from this database as needed for user guidance, real-time visualization, and operational analytics. Through this structure, the system distinctly separates the real-time event-processing path from the data accumulation path, enabling the consistent validation of operating strategies regardless of the demand source.

**Fig 1:**
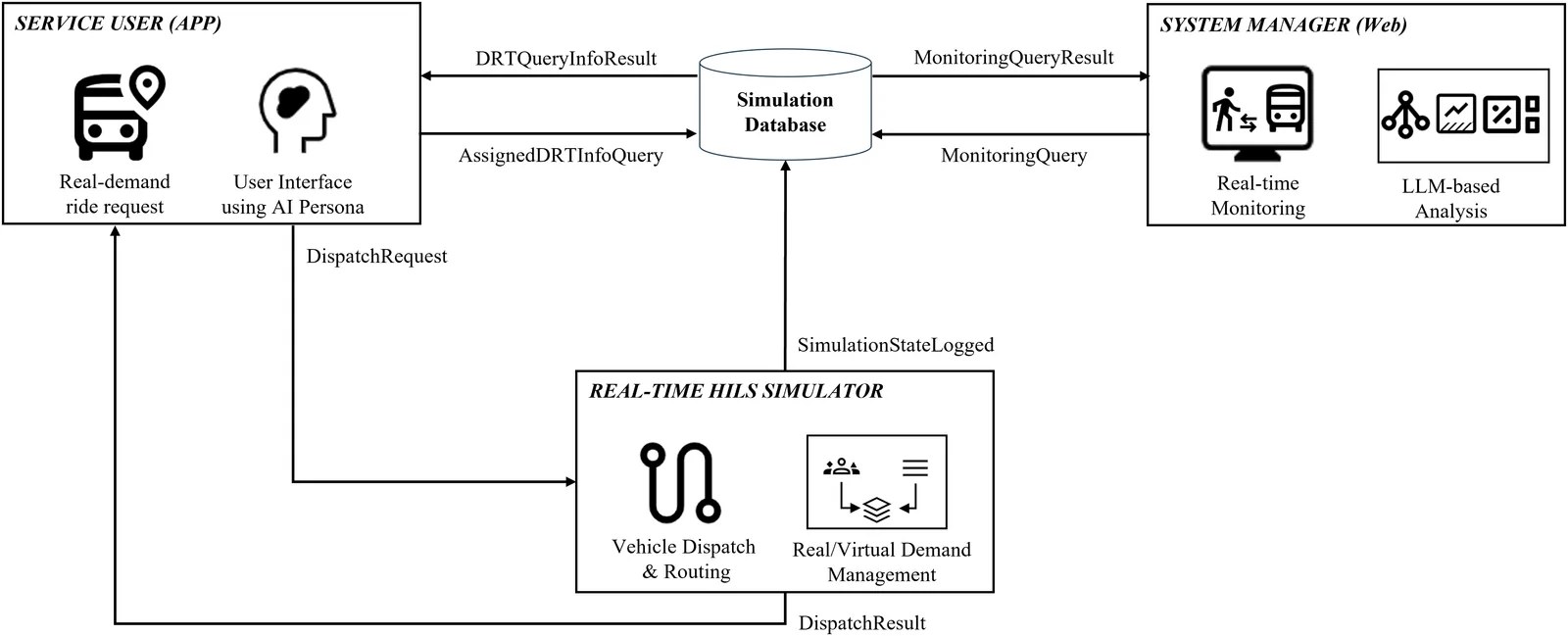
Overall system architecture. (Note: Icons used in this diagram are sourced from Google Material Symbols under the Apache License 2.0.).

#### 3.1.2 Service user (App) architecture.

Fig 2 illustrates the architectural design of the Service User layer in the proposed framework. Moving beyond a simple user interface, the Service User system treats user-entered DRT requests as externally submitted demands and transmits them to the Real-time DRT Simulator through a real-time data transmission channel. The simulator sends dispatch decision results, such as dispatch success or failure, assigned vehicle, and estimated time of arrival (ETA), back to the app backend, which then notifies the user. Consequently, the entire process, from request generation and simulation-based dispatch processing to the result notification, is seamlessly integrated. This ensures that the outcomes of the operational logic are immediately reflected in the user interface, including waiting times, assignment status, and guidance screens.

**Fig 2:**
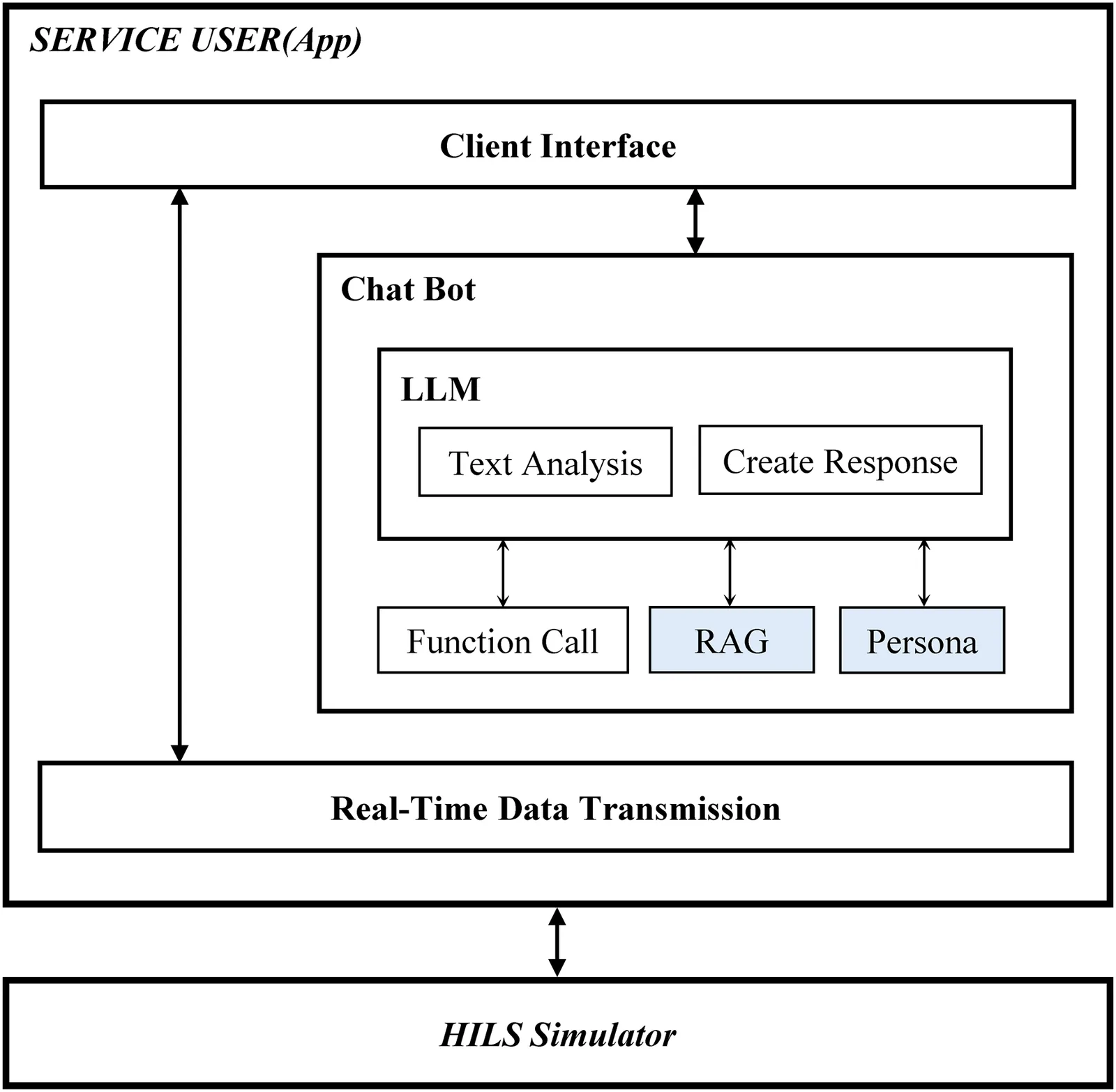
Service User architecture.

The DRT request service within the app is implemented as a hybrid input process consisting of a structured map-based interface and a persona-based natural language chatbot. In the map-based interface, riders can manually search for or select their origin and destination on the map, and the selected latitude and longitude coordinates are mapped to the corresponding candidate stop node or nearest network node before being converted into structured simulator input. In the chatbot interface, riders describe their trip request in natural language, and the LLM-based function-calling module converts the request into a JSON-based dispatch request containing structured fields such as the origin, destination, passenger count, and spatial information. Regardless of the input path, the extracted or selected request information is visualized on the map and presented to the rider for confirmation. After confirmation, the request is converted into a common structured request format and transmitted to the Real-time DRT Simulator.

In addition, the real-time communication channel employs a dual structure based on message characteristics. Time-sensitive, event-driven messages, such as dispatch requests and dispatch results, are transmitted directly to and from the Real-time DRT Simulator via TCP/IP sockets to ensure immediate responsiveness. In contrast, continuously updated state data, such as the dispatch status, ETAs, and route information, are shared through a PostgreSQL database so that both the app and simulator can reference consistent data. When the dispatch is successful, the assigned vehicle location and ETA are visually displayed on the map to reduce user uncertainty. When dispatch fails, the system provides a clear explanation or guides the user through a flow to submit a new request.

The LLM processing structure of the chatbot is organized as a four-stage modular pipeline comprising Text Analysis, Function Calling or RAG, Persona application, and Response Generation. The core logic of this pipeline is formalized in Algorithms 1–3. Following the Text Analysis stage, the process branches into two paths, depending on the nature of the input. During Text Analysis, the system extracts and parses core parameters, such as the origin, destination, and passenger count, from the user input text to structure the data, whereas the LLM simultaneously determines whether the input corresponds to a DRT ride request. If the input is identified as a DRT request, it is routed to the function-calling path [31,35]. In this path, the LLM autonomously selects and executes external functions, such as the location search, coordinate conversion, and DRT request generation, based on the conversational context, thereby transforming the natural language input into actual API requests [31,35]. If the required information is missing, the chatbot improves conversational reliability through follow-up questions and confirmation flows.

For general questions unrelated to dispatching or request handling, such as service information and FAQs, the input is routed to the RAG path [36,37]. This path retrieves similar documents from a knowledge base in which service rules, stop information, and request-related FAQs are indexed as vectors using a pre-trained sentence embedding model hosted on Hugging Face [49]. Only when the dot-product similarity between the user query vector and document vectors exceeds a pre-defined threshold is the single most similar document inserted into the prompt as contextual information [50]; otherwise, the response is generated solely based on the user query. The knowledge base consists of five documents describing service areas, operating conditions, fleet size, and service characteristics. Finally, both the function-calling and RAG paths pass through a common Persona stage before the response generation. At this stage, a character prompt that defines the tone and personality of a persona is fixed as a system prompt, thereby functioning as an interface element that enhances user satisfaction and service accessibility.

The core logic of the above pipeline is formalized in Algorithms 1–3. Specifically, Algorithm 1 defines the overall response generation flow by selecting either the function-calling or RAG path upon receiving a user message. Furthermore, Algorithm 2 describes the analysis and execution process within the function-calling path, wherein the LLM is provided with function specifications to determine the appropriate function and its arguments and subsequently execute the selected function [35]. Finally, Algorithm 3 outlines the process within the RAG path, where the system either leverages the knowledge base through embedding-based similarity retrieval [49,50] or generates a response in the tone of the persona based solely on the user query.

**Table:** 

Algorithm 1: Chat Response Generation (Chat API)
Input: request_message (user input) Output: response_message (chatbot response) 1: CONTEXT ← CONTEXT ∪ {role: “user,” content: request_message} 2: analyzed_dict ← Analyze(request_message, func_specs) // LLM decides if function call is needed 3: if analyzed_dict.function_call ≠ null then 4: response ← Run(analyzed_dict, CONTEXT) // execute function and get result 5: CONTEXT ← CONTEXT ∪ {role: “assistant,” content: response} 6: response_message ← GetResponseContent(CONTEXT) 7: HandleTokenLimit(CONTEXT) 8: return response_message 9: else 10: response_message ← MakeResponseRAG(request_message) // RAG path 11: return response_message 12: end if
Algorithm 2: Function Calling (Analyze and Run)
Input: user_message, func_specs (list of function specifications) Output: message_dict (contains function_call or content) 1: messages ← [system_prompt, {role: “user,” content: user_message}] 2: response ← LLM.chat(messages, functions ← func_specs, function_call ← “auto”) 3: return response.choices[0].message // Run: execute the chosen function and form the response Input: analyzed_dict, context (conversation history) Output: response (function result or LLM-generated response) 1: func_name ← analyzed_dict[“function_call”][“name”] 2: func_args ← Parse(analyzed_dict[“function_call”][“arguments”]) 3: func_response ← Call(func_name, func_args) // search_route, confirm_route 4: context ← context ∪ {role: “function,” name: func_name, content: func_response} 5: return FormatResponse(func_response) // this system returns function output as response
Algorithm 3: RAG-based Response Generation
Input: message (user query) Output: response_message (chatbot response) 1: D ← knowledge base document set (texts) 2: E ← EmbeddingModel 3: q_vec ← E.embed_query(message) 4: D_vecs ← E.embed_documents(D) 5: similarity ← q_vec· D_vecs^T // dot product (per-document similarity) 6: sorted_idx ← argmax(similarity) // index of most similar document 7: if similarity[sorted_idx]> θ then // θ = 150 8: retrieved ← D[sorted_idx] 9: prompt ← CharacterPrompt + “Rephrase the following in the character’s tone: “ + retrieved 10: else 11: prompt ← CharacterPrompt + “Respond to the following in the character’s tone: “ + message 12: end if 13: response_message ← LLM.generate(prompt) 14: return response_message

#### 3.1.3 System manager (Web) architecture.

The System Manager is an operator-facing monitoring and analytical interface designed to enable operators to observe operational conditions in the real-time simulation environment and analyze large volumes of log data. To distribute the processing load of the system and allocate the most appropriate pipeline based on the nature of the information, the proposed architecture operates with a dual structure: a dashboard update flow that performs periodic status updates and a chatbot request flow that processes the unstructured natural language queries of operators. Fig 3 shows a logical sequence diagram of these two core processes within the System Manager layer.

**Fig 3:**
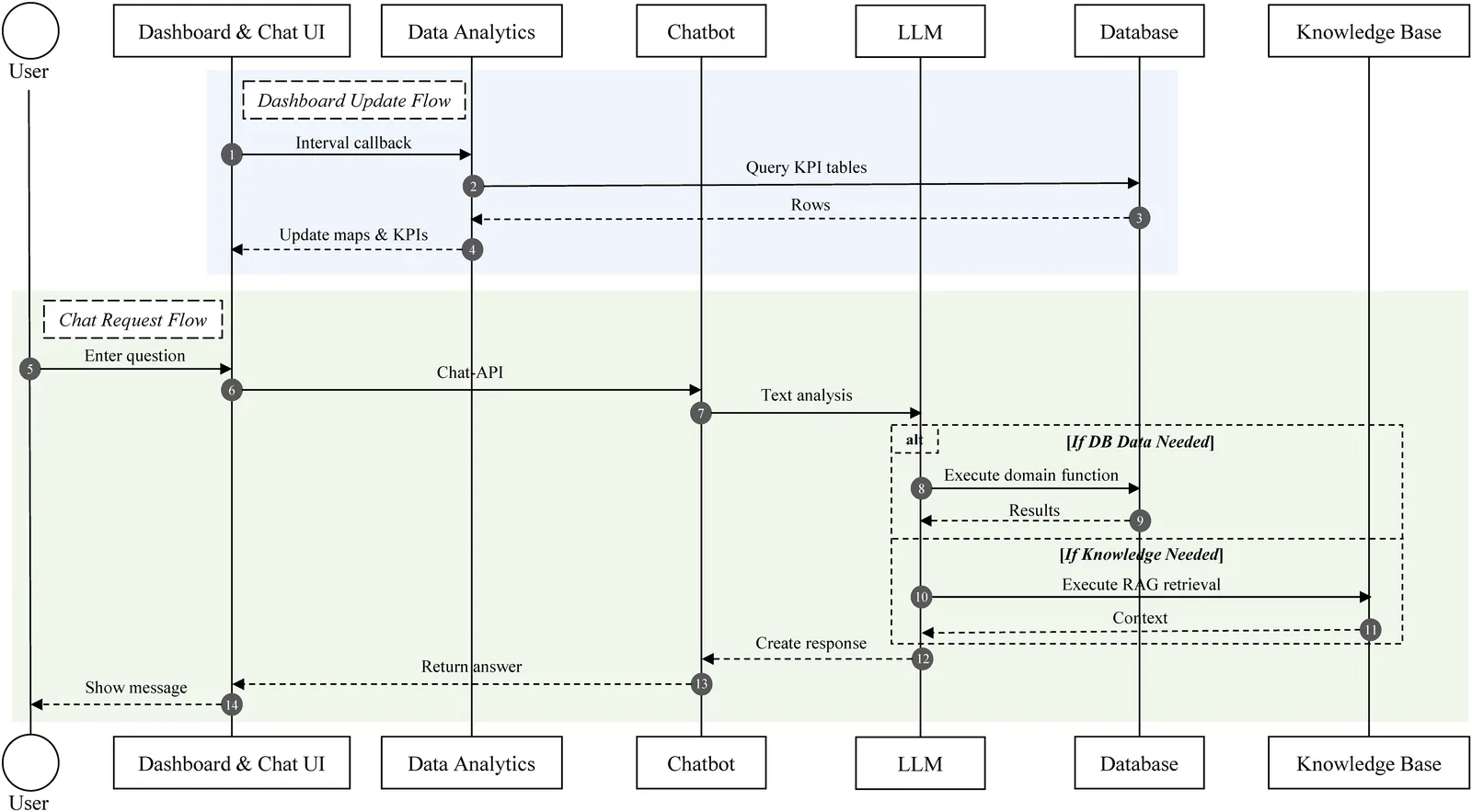
System Manager (Web) logical sequence diagram.

To support real-time state visualization and natural-language-based operational analysis, the proposed System Manager combines a real-time visualization pipeline with an LLM-based analytical pipeline. The Real-time DRT Simulator records discrete-event logs and KPI data in a relational database. At pre-defined intervals, the web client requests status updates from the backend server, which retrieves the relevant data from the database and returns them to the client. The retrieved data are then used to update map-based location markers and quantitative charts, thereby maintaining synchronization between the simulation timeline and the operator dashboard.

Concurrently, an LLM-based intelligent analysis pipeline is configured to process operator queries. The natural language queries of operators are delivered to the system through the API layer; during this process, a mapping layer is first applied to convert system-level node IDs in the database into semantic labels, thereby minimizing semantic ambiguity during the interpretation of the language model. The LLM controller then classifies the intent of each query by dynamically selecting either a quantitative analysis or knowledge-retrieval-based response path according to the characteristics of the query.

For queries that require quantitative statistics or arithmetic operations, the LLM is constrained to function not as a free-form generator, but as a trigger for function calling to ensure response accuracy [31,35]. Specifically, the LLM analyzes natural language queries and invokes pre-defined domain-specific computational functions [35], whereas the internal computation module directly executes SQL queries on the database to produce statistical results. The system constructs a final response based on these results; this layered separation mitigates the risk of numerical errors caused by language model hallucinations [51,52].

By contrast, for queries involving unstructured domain knowledge, the RAG path is applied [36,37]. The system vectorizes the input query, retrieves and extracts document fragments from the knowledge base that satisfies the similarity criterion, and injects them into the prompt as contextual information to generate a response. This process enhances the domain relevance and reliability of the final response [37,53].

Therefore, the System Manager should be interpreted as an operator-assisted information-access and analytical interface rather than an autonomous diagnostic or closed-loop control module. Its current functions are limited to translating natural-language queries into pre-defined operations for data retrieval, KPI computation, and vehicle-status monitoring. It does not automatically identify spatial bottlenecks, detect localized service failures, or infer their causes. In addition, the current implementation does not allow operators to modify dispatching policies or feed operational decisions back into the simulator during execution.

Accordingly, the localized delay burden observed in Experiment 3 was revealed through separate post-experimental analyses of the target, background, and overall passenger groups, rather than through automatic diagnosis by the System Manager. Extending the System Manager to support autonomous bottleneck detection would require additional diagnostic logic and comparative functions that evaluate operational indicators across passenger groups, spatial areas, and temporal windows.

#### 3.1.4 Real-time DRT simulator architecture.

The Real-time DRT Simulator is a core layer designed to directly couple request events arising through the Service User interface with a simulation execution loop, thereby enabling the validation of DRT operating strategies under real-time simulation conditions. As shown in Fig 4, it comprises a four-layer internal structure: an External Interface, Real-time Discrete Event Simulation Engine, Simulation Model, and Algorithm.

**Fig 4:**
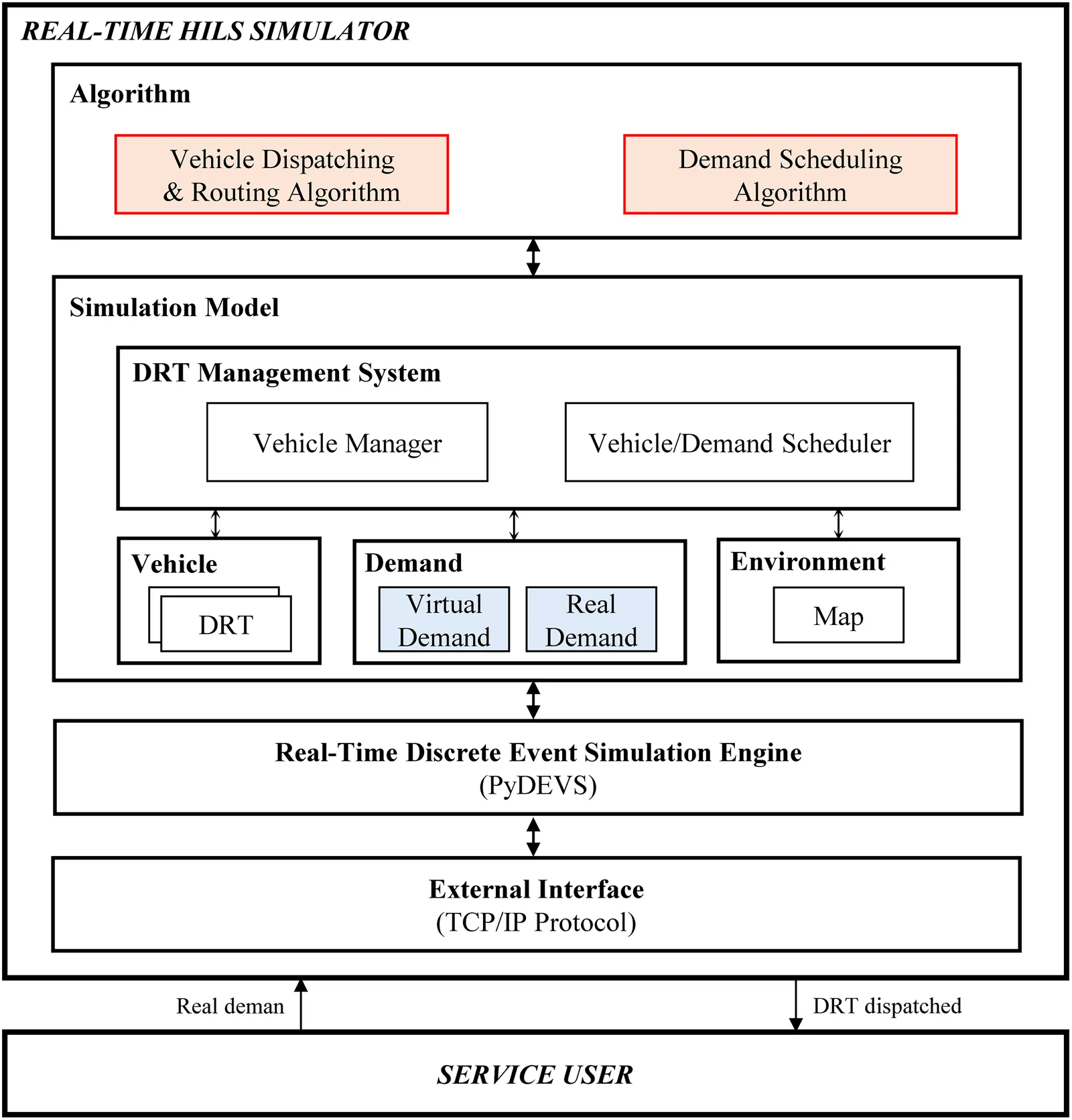
Structure of the real-time DRT simulator.

First, the External Interface (TCP/IP Protocol) is responsible for real-time communication with the Service User layer. It immediately delivers requests submitted by users through the Service User interface as internal simulator events and returns the dispatching and routing results computed by the simulator back to the user layer. Unlike offline log-based experiments, this interface layer assumes that external inputs arrive continuously during simulation execution. Therefore, it is designed to reliably handle the reception of request events and the transmission of response messages. To reduce coupling with internal simulator modules, communication is based on a standardized message structure, including the request ID, occurrence time, origin and destination, stop information, and passenger count.

Next, the Real-time Discrete Event Simulation Engine employs the PyDEVS engine based on the DEVS formalism [39,40,54] to schedule major actions occurring in DRT operations at the discrete-event level and execute state transitions and time advancement. Running on this engine, the Simulation Model is configured to include both the physical components of the DRT system, such as vehicles and passengers, and the management system that operates them. More specifically, the upper level of the simulation model contains the DRT Management System, which is internally divided into the Vehicle Manager and the Vehicle/Demand Scheduler. The Vehicle Manager oversees the vehicle operating status and current location, whereas the Vehicle/Demand Scheduler governs the demand registration and state management (waiting, assigned, boarded, and completed), as well as dispatching policies under service rules, such as maximum waiting time and allowable detour limits.

From the perspective of lower-level entities, the simulation model consists of Vehicle, Demand, and Environment. The Vehicle serves as the execution entity that generates movement, stopping, boarding, and alighting events for DRT vehicles. Meanwhile, the Environment provides a map- and link-node-based movement space that enables the calculation of vehicle travel time and distance, as well as route execution.

The demand generation mechanism is specifically designed to integrate externally submitted and simulation-generated demands within a unified event structure in accordance with the objectives of this study. Fig 5 presents the DEVS diagram [38,39] of the Generator atomic model, which is responsible for generating externally submitted demand from user requests entering through the External Interface, as well as simulation-generated demand from a pre-defined demand model within the simulation. The Generator model consists of three states: Virtual GEN, Real GEN, and SIM DONE. In the Virtual GEN state, simulation-generated demand is generated according to pre-defined interarrival intervals, and the time advance function is defined as $ta=t_{Gen}$. By contrast, in the Real GEN state, events are designed to occur immediately upon receipt of externally submitted user requests through the external interface; therefore, the time advance function is set to ta=0. This ensures that external inputs are converted into internal events without delay at the exact moment that they occur. The SIM DONE state is defined as a terminal state in which no additional demand is generated, and a time advance of ta=∞is assigned to prevent any further internal events.

**Fig 5:**
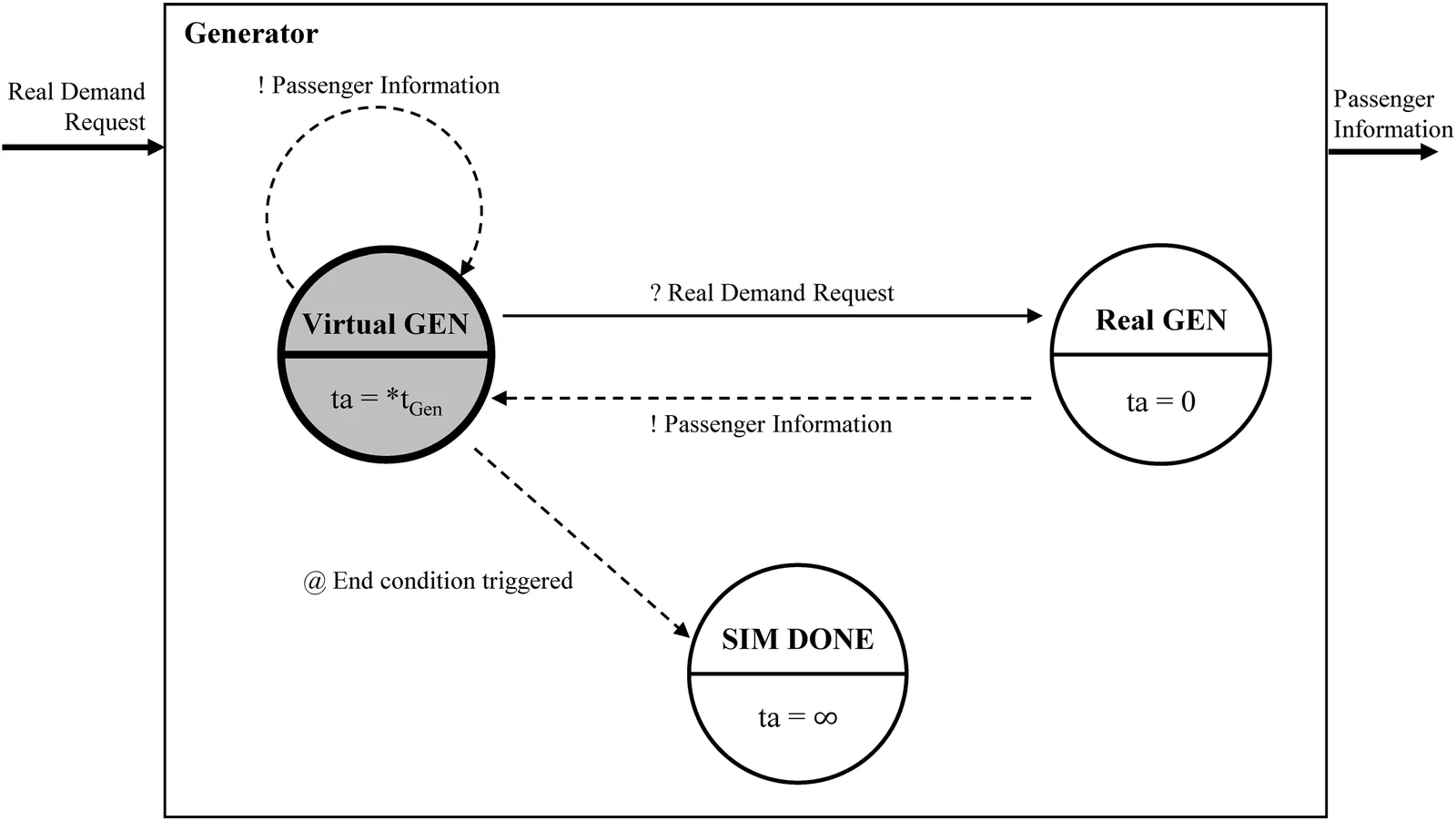
DEVS diagram of the generator.

Importantly, although the Virtual GEN and Real GEN states differ in their generation paths, the resulting demand objects share the same data structure and are delivered to the upper-level DRT Management System through identical ports and event types. Consequently, the same state transition functions and dispatching logic are applied regardless of the origin of the demand. This enables a consistent comparison and validation of the dispatching policies and algorithm performance, even in environments where externally submitted and simulation-generated demands coexist. By exploiting the modular characteristics of DEVS formalism [39,40,54], this design clearly separates the demand-generation layer from the operational decision-making layer while achieving integration at the event level.

Finally, the top-level Algorithm layer contains the core decision-making logic for the simulator execution and is functionally divided into the Vehicle Dispatching and Routing Algorithm and the Demand Scheduling Algorithm. A key advantage of this structure is the clear separation of the algorithm layer from the simulation model and the engine. This modularity allows different algorithms to be seamlessly interchanged while preserving an identical simulation environment and event-processing mechanism. Consequently, the Real-time DRT Simulator provides an integrated experimental foundation for validating DRT operating strategies in a realistic execution context. By jointly processing externally submitted and simulation-generated demands, the simulator ensures real-time handling of external user requests through its interface, whereas the discrete-event engine guarantees stable, event-driven time progression [38,39,55,56].

### 3.2 Proposed system implementation

This section describes the implementation of the proposed framework and the underlying technology stack. The system was developed across the three core layers (Service User, System Manager, and Real-time DRT Simulator), with each layer tailored to translate the designed information flow and functional architecture into a fully operational environment. The following subsections describe the specific implementation details and major software components corresponding to each of the three layers.

#### 3.2.1 Service user (App).

This subsection details the implementation of the Service User layer and its specific technology stack, focusing on the human–system interactions through which users submit DRT requests. The client application was developed using the Flutter framework (Dart) and the chatbot module was implemented using the LangChain framework [57] coupled with OpenAI GPT-4o-mini-API [31]. For the RAG module, a pre-trained sentence embedding model hosted on Hugging Face was utilized [49]. Furthermore, a dual-communication architecture was realized using TCP/IP sockets for time-sensitive event-driven message transmission, along with a PostgreSQL database for state data sharing.

Fig 6 presents an example interface demonstrating a human–system interaction session through the natural-language chatbot. Through natural language conversations, users can input their origins and destinations. Once the chatbot extracts and summarizes the collected information, the user reviews it and confirms the details. The confirmed location data are then converted into geographic coordinates via function calling [35] and immediately visualized as markers on the map interface. This seamless integration of the chatbot and map interface consolidates natural language input and spatial visualization into a unified request generation process.

**Fig 6:**
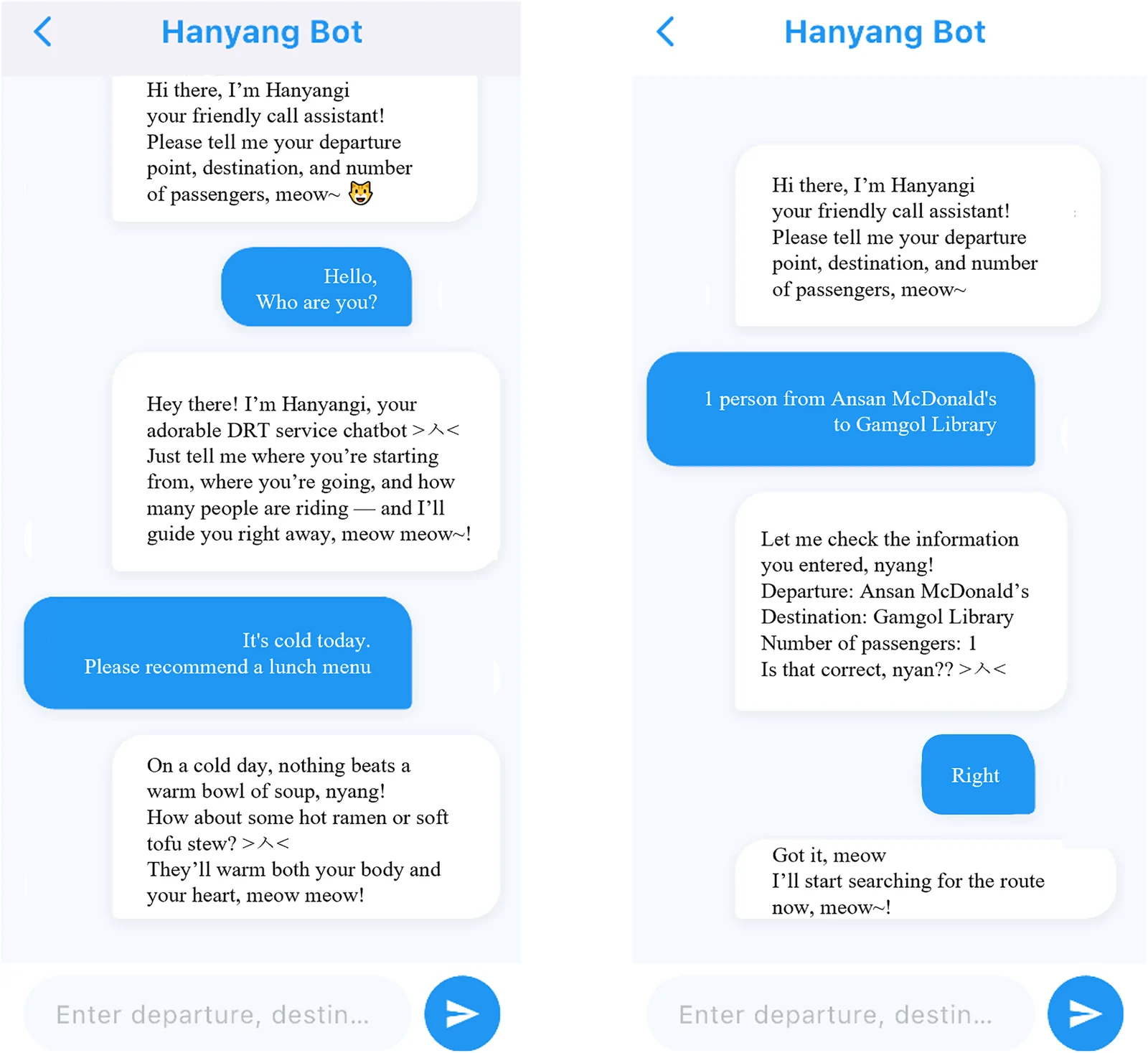
Natural-language chatbot interaction for DRT request input.

Fig 7 illustrates the implementation of the Service User pipeline within the actual Service User interface. Specifically, this sequence demonstrates that the request generation process comprises stepwise human–system interaction: input, verification, confirmation, and result notification. The final output is returned as spatiotemporal information, including the assigned route and ETA. This sequence illustrates that the proposed system operates not only as a text-based response interface, but also as a practical DRT operation framework that seamlessly integrates map-based spatial visualization with the outcomes of vehicle dispatching and routing.

**Fig 7:**
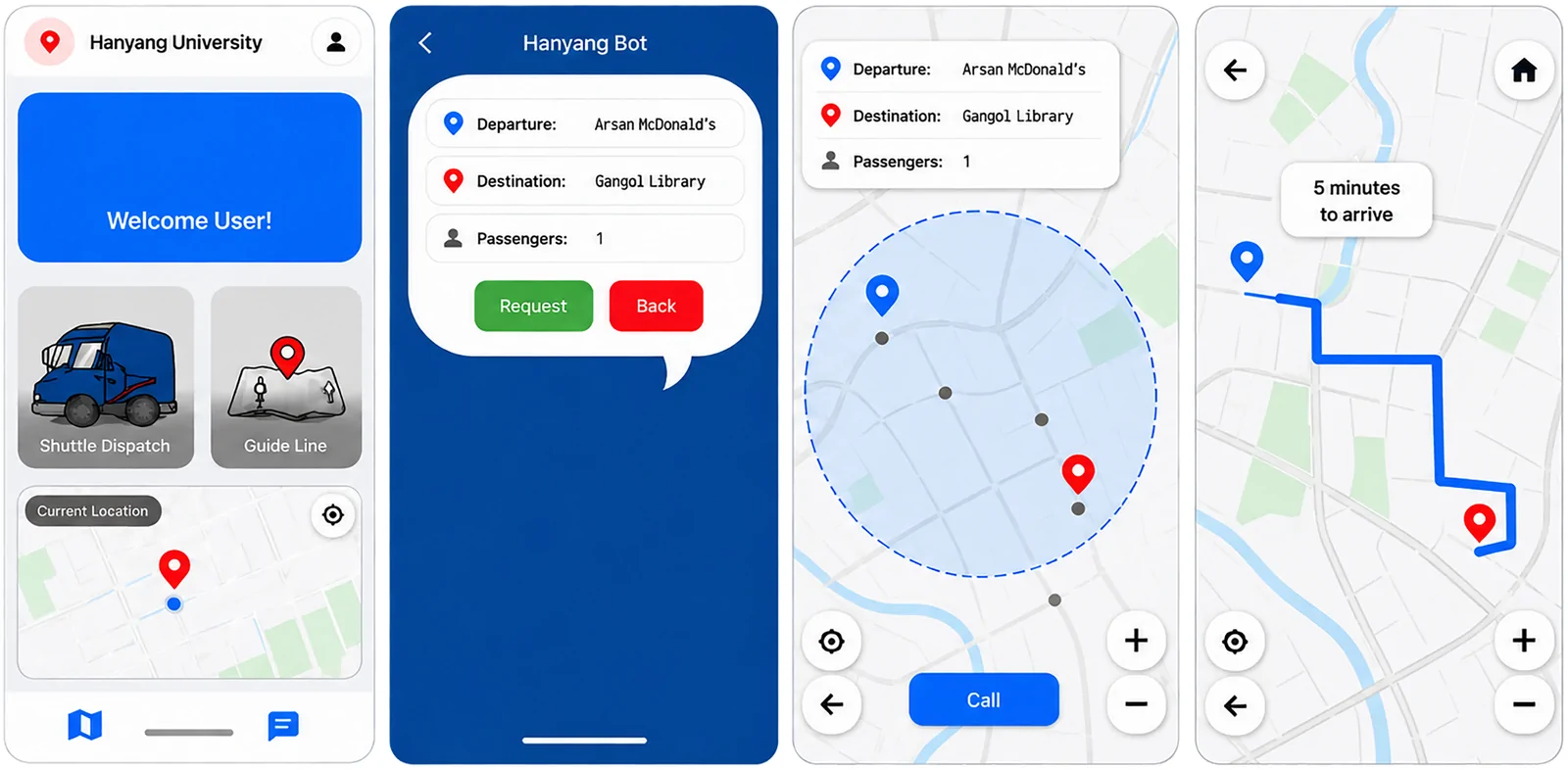
End-to-end DRT service workflow in the service user (App).

The interface sequence unfolds through the following four stages: (a) On the main screen, the user receives service guidance and accesses the interface for DRT requests. (b) Through either the map interface or the natural language chatbot, the user inputs the origin, destination, and passenger count; these selected locations are immediately reflected as departure and arrival markers on the map, allowing the user to visually confirm their spatial validity. (c) Before the request submission, the system presents a summarized review of the core parameters (origin, destination, and passenger count), which enables the user to execute the final confirmation of the DRT request. (d) Upon successful vehicle dispatch, the interface visually displays the route and ETA of the assigned vehicle on the map. This final stage confirms that dispatching outcomes are communicated not only as binary completion statuses but also as concrete spatial (route and location) and temporal (ETA) data.

#### 3.2.2 System manager (Web).

To implement the previously designed System Manager architecture, this study adopted a decoupled service architecture in which the front end and inference server operate independently. The visualization and monitoring interface of the System Manager was built using the Dash framework (Python) and the Plotly library. By contrast, the inference server, which is responsible for the intelligent analysis pipeline, was implemented as a standalone REST API server based on the Flask framework. These two components communicate via HTTP requests with a PostgreSQL relational database serving as the centralized repository for the core data of the system. Fig 8 illustrates the comprehensive interface of the implemented System Manager and the configuration of its five core functional modules.

**Fig 8:**
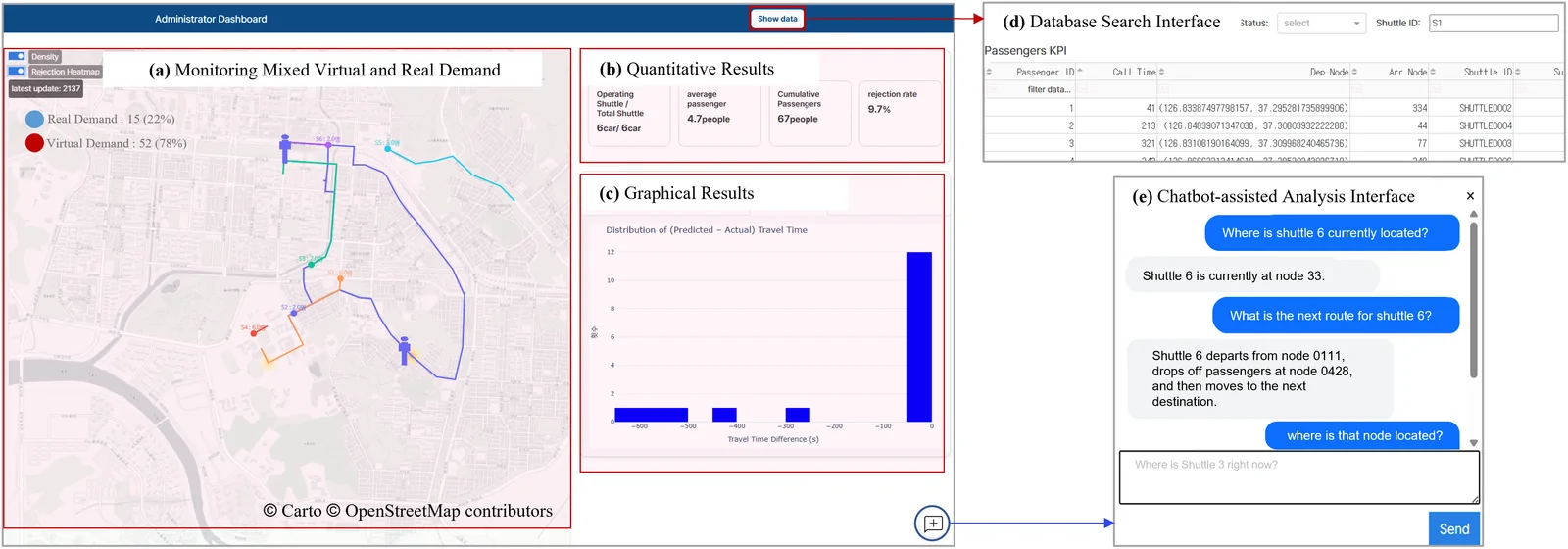
Configuration of the proposed system manager (Web) integrated monitoring and analysis dashboard.

In module (a) on the left side of Fig 8, the integrated monitoring map for externally submitted and simulation-generated demands is implemented using a Plotly Scattermapbox object. By projecting externally submitted and simulation-generated demands onto a map with distinct colored markers, this module enables operators to intuitively recognize complex demand concentrations. In the upper-right area, modules (b) the quantitative KPIs panel, (c) visualization-based analytical charts, and (d) the database search interface are updated via a periodic polling mechanism using the Dash dcc.Interval component. To reduce the excessive load on the database server caused by frequent updates, the system employs a memory-based temporary storage structure. Specifically, the vehicle and passenger data retrieved from the database are loaded into the system memory, allowing the dashboard rendering functions to reference these global variables instead of performing direct database queries at every update interval. This design significantly reduces the database access frequency while securing both the responsiveness and stability of the System Manager interface.

In the lower-right area, module (e), the intelligent analysis interface, executes the question-answering pipeline. When a natural language query of an operator is delivered to the inference server through the REST API, a semantic transformation layer is first applied to minimize the interpretive ambiguity. At this stage, the node-conversion module detects system-level node IDs using regular-expression-based boundary matching and replaces them with semantic labels by referencing a pre-defined mapping table. Exception-handling logic ensures that unmapped IDs remain in their original string form to prevent information loss.

The pre-processed text is then delivered to the inference engine for intent classification. Queries requiring quantitative analysis are executed by transforming the input into parameters for pre-defined domain functions rather than allowing the model to generate numerical values directly, which ensures higher consistency and reproducibility compared with unconstrained generation. In contrast, for queries involving unstructured domain knowledge, a RAG pipeline based on LangChain (v0.1.0) and ChromaDB (v0.4.22) is employed. Domain knowledge is converted into high-dimensional vector representations using the all-MiniLM-L6-v2 model [49], with the retrieval process configured to extract the top three candidate documents based on the cosine distance [50]. The implementation applies filtering logic in which retrieved documents are injected into the prompt as context only when their distance values fall below an empirically determined threshold. If the threshold is not met, the system returns a pre-defined template indicating that no relevant information has been found, thereby suppressing hallucinations [51–53]. The final response is returned to the client and displayed on the interface. Through this architecture, operators can query and analyze simulation data states using everyday natural language, eliminating the need for complex SQL queries or manual cross-analysis of multiple charts.

#### 3.2.3 System integration.

Fig 9 illustrates the actual implementation environment of the proposed system. The system consists of three functionally distinct layers (Service User, System Manager, and Real-time DRT Simulator) and is implemented as a distributed execution environment, where each layer operates independently while sharing operational information through a centralized database. This architecture realizes a practical DRT operating environment at the level of an actual system, where request handling, vehicle dispatching, and operational monitoring are connected continuously and seamlessly.

**Fig 9:**
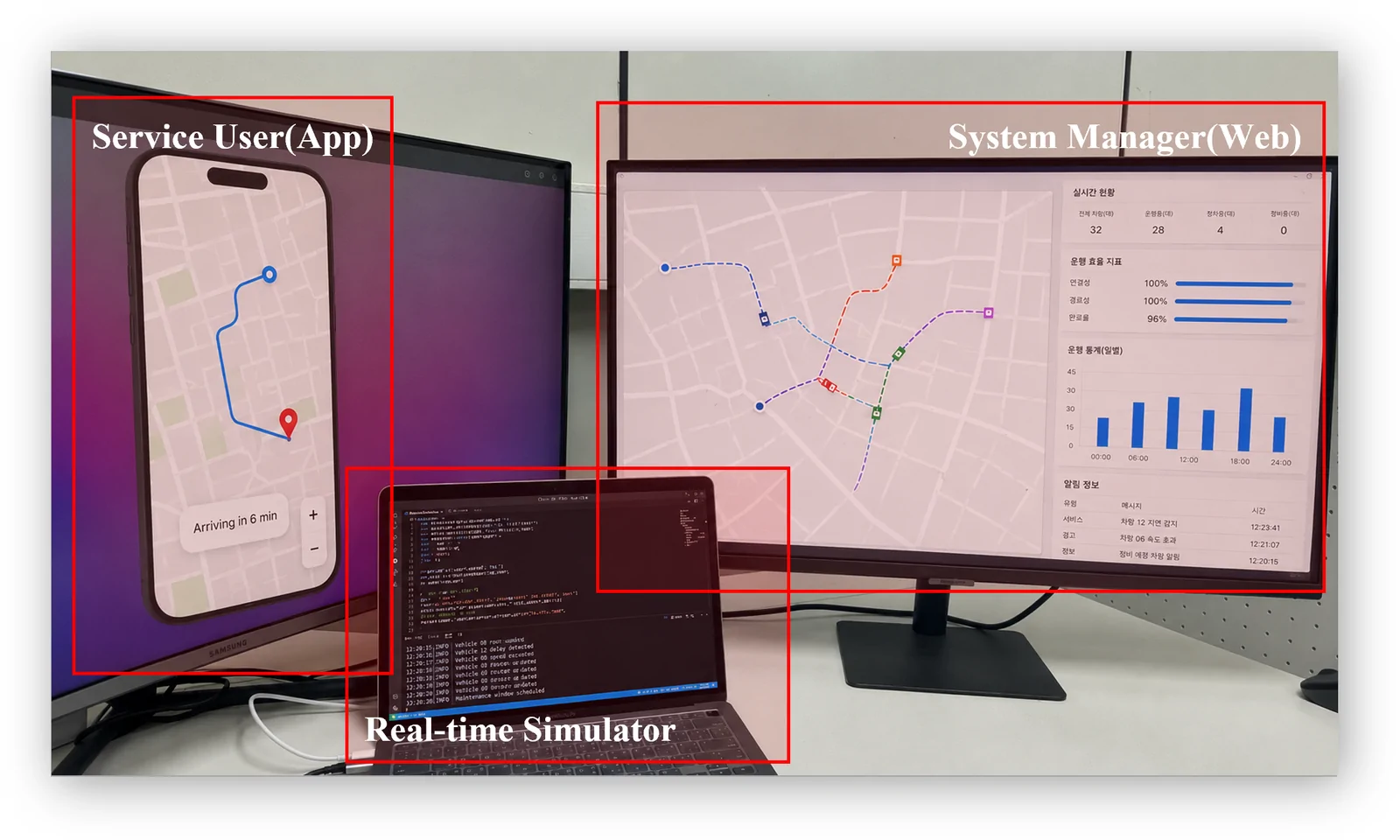
Proposed system implementation.

## 4. Experiments

### 4.1 Experimental design

The experiments were conducted in a DEVS-based real-time simulation environment [38–40]. The simulation components were implemented as atomic models and were designed to interact using coupled models. The experimental space was defined as parts of a transport-disadvantaged area and the surrounding university campus in Ansan, and a node-link graph network consisting of 1,087 nodes and 1,588 links was constructed using Geographic Information System (GIS)-based vector map data. Rather than modeling only fixed bus stops as separate entities, this study defined all 1,087 nodes as candidate pickup and drop-off locations, thereby reflecting the flexible stop-search characteristics of DRT services [58,59]. Within this environment, vehicle movement, passenger requests, and dispatching, boarding, and alighting processes were reproduced as discrete events, mirroring real-world operations.

Six DRT vehicles were deployed in the simulation, and the maximum capacity of each vehicle was set to nine passengers. The boarding and alighting times were fixed at 5 s each. Passenger entities were defined with four states (WAIT, BOARDING, END, and FAIL), whereas vehicles were operated through three state transitions (IDLE, MOVE, and BOARD). During the demand processing, the maximum allowable waiting time for a newly generated passenger was set to 900 s. Additionally, the dispatching logic was subject to constraints that ensured that the insertion of a new passenger would not increase the cumulative waiting time for already waiting passengers or the in-vehicle travel time for already boarded passengers by more than 600 s in either case.

Routing was performed using the shortest-path method based on the link travel time as the edge weight. To improve computational efficiency, the shortest-path travel times for all node pairs were pre-loaded at the start of the simulation. For vehicle dispatching, a capacity-constrained rule-based greedy insertion algorithm was applied [60]. Candidate vehicles were first filtered according to their current onboard passenger count and the number of newly requested passengers, ensuring that only feasible vehicles were considered. Next, the Fréchet distance between the origin-destination route of the passenger and the current route of each vehicle was calculated [61,62], and the three vehicles with the smallest Fréchet distances were selected. All possible combinations for inserting the pickup and drop-off points of a new passenger into the existing destination lists were then explored. The insertion position that minimized the total travel time while satisfying all constraints was selected. Finally, the assigned vehicle was determined by calculating an exponential-decay-based satisfaction function that integrated new-passenger, onboard-passenger, and waiting-passenger satisfaction, with weights of 0.4, 0.3, and 0.3, respectively.

Passenger demand was generated to reflect both temporal and spatial characteristics using station-level public transit usage data. Station-level boarding and alighting volume data for the selected stops within the study area were collected from the Transportation Card Big Data Integrated Information System (TCBIS; Korean: 교통카드빅데이터 통합정보시스템), specifically from the “station-level usage volume” indicator under the route and station indicators category. These records were used to construct the temporal and spatial demand distributions for the simulation.

Temporal demand was modeled based on a non-homogeneous Poisson process (NHPP) [63], where the mean arrival rate was estimated from the observed proportion of passenger occurrences in each time period. Specifically, boarding and alighting volumes were aggregated by time period across the selected stops and normalized to obtain relative request-generation ratios. Inter-arrival times of DRT requests were assumed to follow an exponential distribution to reproduce time-dependent demand concentration patterns. Spatial demand was generated using a weighted sampling method, in which passenger origins were sampled according to station-level boarding volumes and destinations were sampled according to station-level alighting volumes for the corresponding time period. To reflect uncertainty in origin input that may arise in real-world DRT requests, controlled spatial noise was added by applying a uniformly distributed random jitter to the origin coordinates. The overall simulation parameters used in this study, including the environment, operational resources, dispatching algorithm, and demand-generation method, are listed in Table 2.

**Table 2:** Simulation parameters and operational settings.

Category	Description
Experimental environment	DEVS-based real-time simulation environment with a GIS-based node-link network – 1,087 nodes / 1,588 links
Operational resources	Six DRT vehicles (capacity of nine passengers per vehicle), with 5 s for boarding and alighting
Routing method	Shortest-path routing based on link travel time
Dispatching method	Capacity-constrained greedy insertion dispatch [60]
Demand generation	NHPP-based temporal demand [63] and weighted spatial demand

To align with the three-layer architecture proposed in this study, the experimental design comprised three phases. Experiment 1 evaluated the natural language interface of the Service User layer; Experiment 2 assessed the intelligent analysis interface of the System Manager layer; and Experiment 3 investigated system-level response characteristics within the Real-time DRT Simulator. Essentially, Experiments 1 and 2 validated the user- and operator-facing interfaces, an aspect relatively underexplored in previous DRT studies, whereas Experiment 3 examined operational vulnerabilities in the real-time simulation environment that remain obscured during conventional aggregate-level evaluations based on simulation-generated demand.

Table 3 lists the target systems, experimental objectives, baseline comparisons, and evaluation metrics of the three experiments. The experiments were broadly categorized into functional validation and system-level stress testing. The functional validation stage (Experiments 1 and 2) examined whether the proposed LLM-based interfaces could accurately and rapidly convert the user and operator inputs into structured data for system processing. By contrast, the stress-testing stage (Experiment 3) evaluated whether the Real-time DRT Simulator, together with post-experimental analysis, could reveal operational bottlenecks and delay propagation under real-world geographic constraints and synchronized demand conditions. The former evaluated component-level accuracy and responsiveness, whereas the latter assessed the analytical utility of the framework for real-time operations.

**Table 3:** Overview of the experimental setup and evaluation metrics.

Experiment	Target System	Experimental Objective	Experimental Method	Evaluation Metrics
1	Service User	Functional verification of LLM-based request parsing	Functional verification using 100 pre-defined natural-language requests, with GUI-derived coordinates and passenger counts used as ground-truth references	Exact-match accuracy of origins, destinations, and passenger counts against GUI-derived ground-truth values; response latency
2	System Manager	Functional verification of the function-calling-based manager query path	Functional verification using 100 pre-defined operator queries, with direct SQL results and dashboard metrics used as ground-truth references	Exact-match accuracy against ground-truth values and response latency
3	Real-time DRT Simulator	Stress test under context-based synchronized demand	Comparative analysis of temporal demand distributions (Uniform, Poisson, and Synchronized) across three spatial scenarios (Festival, Lunch, and Holiday)	Mean wait time, P95 wait time, ECDF, Gini coefficient, and wait burden share (Target vs. Background vs. All passengers)

### 4.2 Experimental results and discussion

Traditional simulation-based evaluations often model vehicle movement and dispatch logic in detail; however, they frequently fail to adequately capture interface-level processing stages, such as the user interface operation time, operator query processing time, and communication delays between system components. Therefore, the functional validation experiments (Experiments 1 and 2) treated these interface components as independent evaluation targets. These experiments aimed to examine how accurately and rapidly the LLM can transform unstructured natural language inputs from users and operators into structured, system-processable data (e.g., coordinates, dispatching parameters, and computational queries). Ultimately, these functional validations empirically addressed a critical gap in previous DRT studies [7–9,27], which have largely marginalized the user- and operator-facing interface functions within the Service User and System Manager layers.

#### 4.2.1 Experiment 1: Verifying the user natural language call interface functionality.

In DRT environments, fixed routes are absent and stopping locations operate flexibly, implying that multiple points can serve as potential pickup and drop-off candidates [58,59]. Consequently, users often experience cognitive burdens and operational delays when directly searching and pinning accurate origins and destinations on a smartphone map interface. To address this, Experiment 1 evaluated whether the natural language interface of the Service User layer could accurately and rapidly convert unstructured natural language requests into structured data (i.e., stop nodes, coordinates, and passenger counts) required by vehicle dispatching and routing logic. To establish ground-truth reference values, a map-based graphical user interface (GUI) was utilized in which users manually specified locations via zooming and scrolling. Rather than serving as a formal control group for usability testing, this GUI setting provided the ground-truth reference coordinates and parameters necessary to assess the accuracy and consistency of the LLM natural language parsing.

To reflect the diversity and ambiguity of natural-language requests involving location recognition, the test scenarios were organized into six categories, comprising a total of 100 cases. These categories included single-destination inputs, explicit origin–destination pairs, requests combining passenger counts with location information, landmarks or ambiguous expressions using business names or informal references instead of official stop names, typographical errors, and linguistic variations expressing the same travel intent in different ways. Representative examples included “Take me to Gamgol Library” for a single-destination input; “I want to go from ERICA Main Gate to the dormitory” for an origin–destination pair; “Two of us are going to the main gate, so please send a car” for a request including passenger count; “Please pick me up in front of the McDonald’s in Gojan-dong” for an ambiguous landmark expression; “Let’s go to Gamgol Libary” for a typographical error; and alternative phrasings of an identical trip request for the linguistic-variation category.

Fig 10 illustrates the Service User interface implemented for the functional validation. The evaluation metrics were accuracy and latency. The accuracy was determined by verifying whether the coordinates and passenger counts returned by the LLM exactly matched the GUI-derived reference values across all query categories. The latency was measured as the time elapsed from the text submission by the user to the successful generation of structured request data by the system, evaluating the viability of the interface for real-time DRT operations.

**Fig 10:**
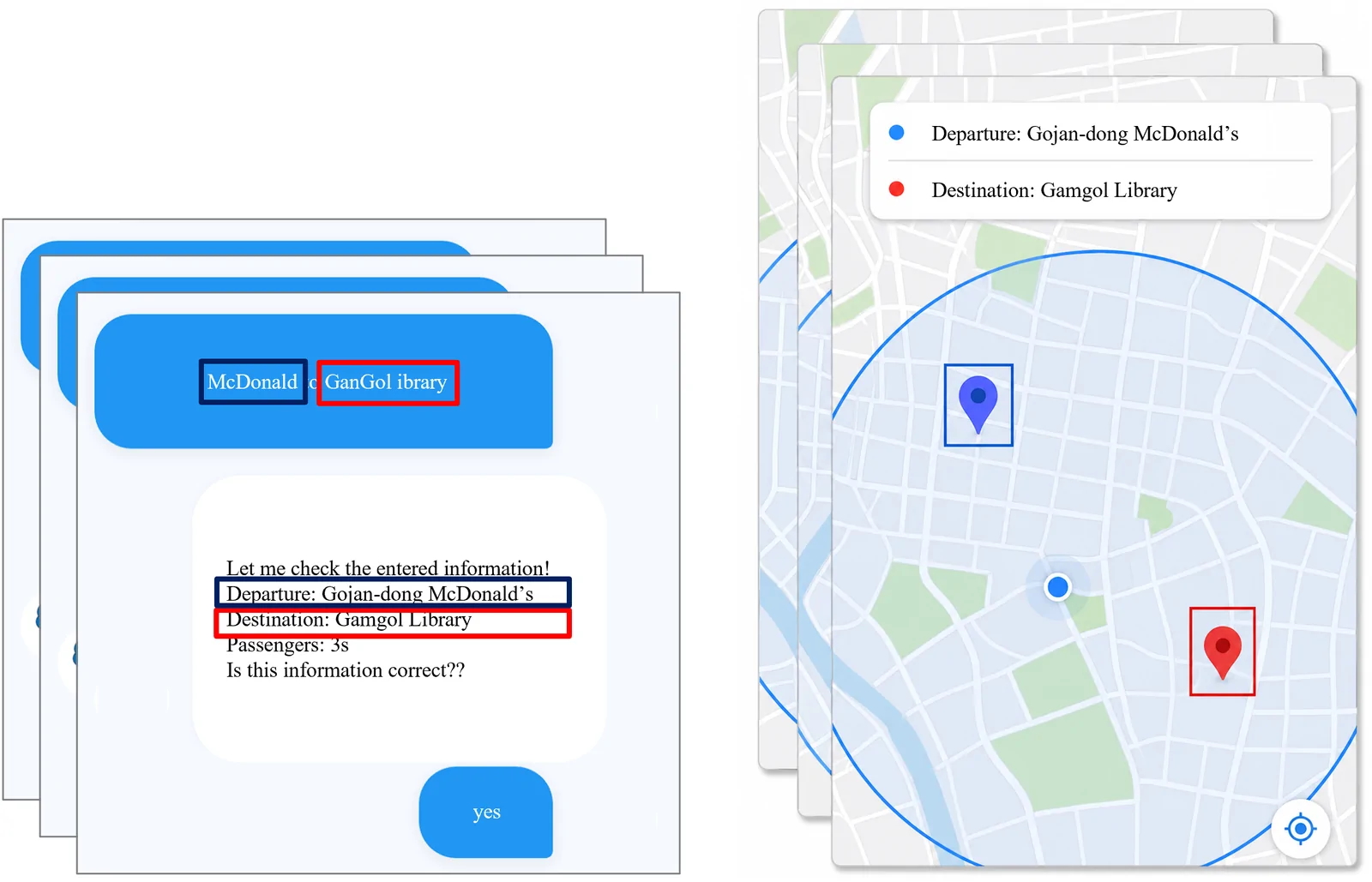
Implementation of the proposed system interfaces for functional verification.

The evaluation results demonstrate that the proposed LLM interface successfully extracted the stop-node coordinates and passenger-count parameters required by the vehicle dispatching and routing logic across all query types without omission. Table 4 lists the LLM parsing accuracy and average processing latency across the six natural language query categories. For the entire 100-case test set, the parsing results achieved 100% exact-match accuracy against the ground-truth reference values within the pre-defined test set. These results confirm that, within the pre-defined test set, the LLM-based interface could process diverse unstructured natural-language requests, including landmark-based expressions, typographical errors, and alternative phrasings, and convert them into the structured parameters required for dispatching. Because a rule-based parser was not included as a comparison baseline, the present experiment does not establish the superiority of the LLM-based approach over rule-based or keyword-matching methods. Nevertheless, because these results were derived from controlled functional validation using pre-defined scenarios, they do not guarantee generalization to open environments. Further validation incorporating a wider array of natural language variations, incomplete inputs, and actual human–system interactions is required.

**Table 4:** Functional verification of the User Interface (LLM-based Dispatch).

ID	Category	User Query (Natural Language)	Parsed Action / Coordinates	Status	Latency (s)
1	Single Dest.	“Go to the Library.”	Dest: Library (126.85, 37.29)	Success	1.05
2		“I want to go to the Main Gate.”	Dest: Main Gate (126.82, 37.30)	Success	1.12
3	O-D Pair	“From McDonald’s to Dormitory.”	Dep: McDonald’s (126.83, 37.31) Arr: Dormitory (126.84, 37.29)	Success	1.25
4		“Call a shuttle from Eng. Bldg to Station.”	Dep: Eng. Bldg (126.83, 37.30) Arr: Hanyang Stn (126.86, 37.31)	Success	1.28
5	Passenger Count	“3 people going to the Student Union.”	Arr: Student Union (126.84, 37.29) Passenger: 3	Success	1.15
6		“Pick up 2 passengers at Yangji High School.”	Dep: Yangji HS (126.82, 37.30) Passenger: 2	Success	1.19
7	Landmark	“I’m at Starbucks in front of the school.”	Dep: Starbucks (126.83, 37.30)	Success	1.32
8		“Take me to Lake Park.”	Arr: Lake Park (126.82, 37.29)	Success	1.21
9	Typographical Error	“Go to Dormitry please.” (Typo)	Arr: Dormitory (126.84, 37.29)	Success	1.35
10	Linguistic Variation	“Please send a car to Gojan Station.”	Arr: Gojan Station (126.81, 37.32)	Success	1.29
Avg.	Total	Tested on 100 pre-defined test queries	–	100%	1.22

Regarding latency, the single-destination category exhibited the shortest average response time, whereas the landmark and typographical error correction categories exhibited relatively higher latencies. This increase is attributable to the additional inference load placed on the LLM when processing nonstandard expressions. Nevertheless, the overall average parsing latency remained approximately 1.22 s, indicating technical responsiveness under the tested conditions. Notably, the absence of parsing failures in scenarios involving ambiguous landmarks or typographical errors suggests that, within the controlled test set, the system could contextually interpret locational intent and derive precise coordinates without relying on official stop names. The measured latency indicates the technical responsiveness of the interface; direct usability and task-time comparisons with the GUI remain for future studies.

#### 4.2.2 Experiment 2: Validation of the LLM-based manager query interface.

In actual DRT operations, unforeseen situations, such as sudden localized demand surges or vehicle status changes, occur frequently. During these events, standardized dashboard charts often fail to provide immediate context-specific information, and manual database queries using SQL introduce significant analytical delays for operators. To address this, Experiment 2 evaluated whether the function-calling-based quantitative analysis path of the System Manager interface could accurately and rapidly convert operators’ unstructured natural-language queries into executable data-retrieval and computational requests.

In the experimental setup, operators submitted complex natural language queries (e.g., “Calculate the dispatch rejection rate for the dormitory area over the last 10 minutes”). Manual SQL queries on raw database rows and existing dashboard metrics were used to establish the ground-truth reference values. As in Experiment 1, this reference setting served strictly to verify the computational and retrieval accuracy of the LLM pipeline rather than functioning as a broad usability control group.

To reflect the complexity of operational monitoring, the query scenarios were divided into four categories comprising a test set of 100 cases. These categories included: (1) location and status queries (e.g., “Where is Vehicle 1 right now?”); (2) simple statistical queries for retrieving unprocessed database values (e.g., “How many total passengers boarded today?”); (3) KPI computation and analysis queries requiring internal calculations (e.g., “Calculate the current dispatch rejection rate”); and (4) multi-object composite analysis queries that integrate data across multiple entities (e.g., “Compare the average waiting times of Vehicle 1 and Vehicle 2”).

Fig 11 illustrates the implementation of the proposed system interfaces for the chatbot. The evaluation metrics were accuracy and latency. The accuracy was determined by verifying whether the final numerical and textual responses generated through the function-calling-based analysis pipeline exactly matched the SQL- and dashboard-derived ground-truth reference values. The latency was measured as the time elapsed from query submission to the final response output to assess the viability of the interface for real-time synchronization with the Real-time DRT Simulator and its capacity for rapid operational decision support.

**Fig 11:**
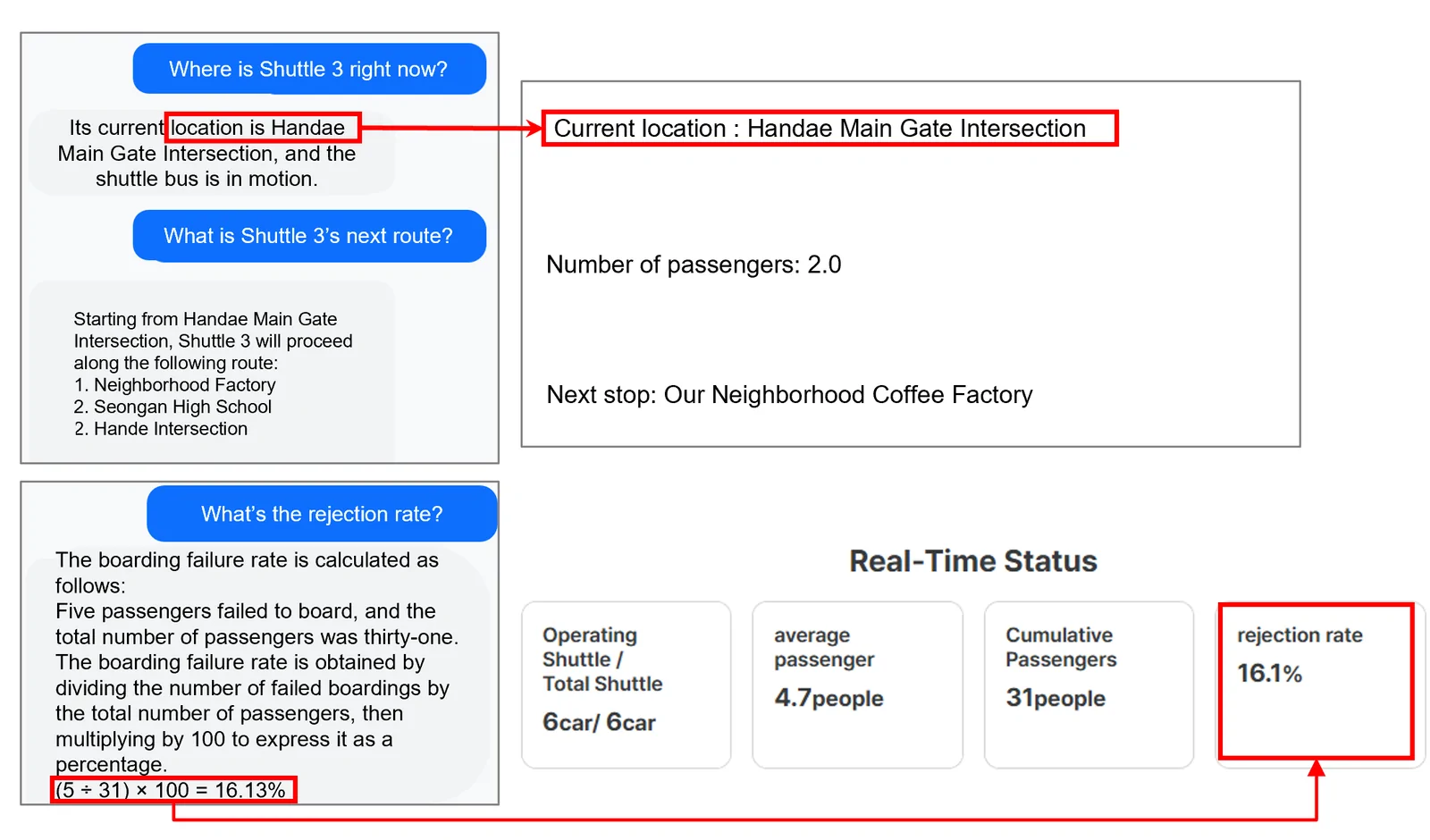
Implementation of the proposed system interfaces for the chatbot. (This web interface is developed using Dash and Plotly, with UI components styled via Bootstrap (COSMO theme)).

The evaluation results demonstrate that the intelligent analysis interface of the System Manager achieved 100% exact-match accuracy across the pre-defined 100-case test set, returning data extraction and computational results that exactly matched the ground-truth reference values for all four query categories. The average latency from query submission to the final response output was 2.21 s. This indicates an acceptable level of responsiveness to real-time operational support under the specific real-time simulation conditions in this study. However, like Experiment 1, these findings reflect controlled validation. Scaling the manager query pipeline to process substantially larger databases or highly concurrent operator requests in a commercial deployment could introduce greater latency overhead, a limitation that necessitates further optimization in future research. Table 5 lists representative examples of actual natural language queries submitted by operators, alongside the corresponding system responses for each query category.

**Table 5:** Functional verification of the System Manager query interface.

ID	Category	User Query (Natural Language)	System Response / Computed Result	Status	Latency (s)
1	Location	“Where is Shuttle #1 right now?”	Loc: Node 33 (Handae Intersection)	Success	1.85
2	Location	“Is Shuttle #3 moving or stopped?”	Status: Moving (Speed: 25 km/h)	Success	1.92
3	Route	“What is the next stop for Shuttle #2?”	Next: Node 428 (Seongan HS)	Success	2.05
4	Route	“Show me the full route of Shuttle #5.”	Route: Main Gate → Dorm → Lib → Station	Success	2.35
5	KPI (Stats)	“How many passengers are on Shuttle #4?”	Pax Load: 5 / 9 (Capacity)	Success	1.98
6	KPI (Stats)	“What is the total number of passengers today?”	Total: 142 passengers	Success	2.10
7	KPI (Analysis)	“Calculate the current rejection rate.”	Rejection Rate: 16.13% (5 failed / 31 total)	Success	2.45
8	KPI (Analysis)	“Which station has the highest demand?”	Top Station: Main Gate (45 requests)	Success	2.55
9	Status	“Are there any idle vehicles?”	Result: Yes, Shuttle #6 is IDLE.	Success	2.01
10	Complex	“Compare the average wait time of Shuttle #1 and #2.”	#1: 7.2m vs #2: 6.8m	Success	2.85
Avg	Total	Tested on 100 pre-defined test queries	–	100%	2.21

Across the full 100-case test set, all four categories produced results that exactly matched the ground-truth reference values (i.e., the raw data obtained via direct SQL queries and the numerical metrics displayed on the dashboard). The most notable demonstration of analytical accuracy occurred in the KPI computation category. For the query “Calculate the current dispatch rejection rate,” the system did not merely retrieve a pre-stored aggregate value; instead, it individually extracted 31 real-time requests and five failed cases, dynamically calculating the dispatch rejection rate as 16.13% (five out of 31 requests). This confirms that the proposed framework performs real-time computations based on the most recent operational data at the exact moment of the query. Furthermore, in the multi-object composite analysis category, the system successfully retrieved data for multiple DRT vehicles, merging and comparing the data within a single response context to yield results that exactly matched the pre-defined ground-truth values.

Regarding processing latency, the average delay from query submission to the final response output was 2.21 s. In the representative cases shown in Table 5, location and status queries required 1.85–2.01 s, whereas KPI and composite analysis queries required 2.45–2.85 s. These results indicate low-second-level functional responsiveness under the prototype conditions. The results demonstrate the functional responsiveness of the manager interface, whereas direct comparisons with manual SQL-based analysis require further user-based evaluation. In conventional operational analysis environments, operators experience significant analytical delays owing to the manual writing of SQL queries, cross-checking of charts, and manual KPI calculations. In contrast, the average response time of 2.21 s supports the technical feasibility of the proposed interface for real-time operational analysis under the prototype conditions.

This experiment verifies the functional correctness and latency of the manager query interface for KPI retrieval, vehicle-status monitoring, and pre-defined quantitative analysis. These functions provide the information-access layer required for operator-assisted interpretation of simulation results. However, autonomous failure diagnosis or automatic bottleneck detection would require additional diagnostic logic beyond the current KPI-query and status-monitoring functions, such as comparisons across passenger groups, temporal windows, spatial clusters, and distributional indicators.

#### 4.2.3 Performance evaluation experiment.

Traditional DRT simulation studies often represent temporal demand variation using probabilistic arrival models such as the NHPP [63]. Although these models are useful for reproducing average temporal demand patterns, they may not capture synchronized demand surges or localized congestion unless such temporal dependence is explicitly incorporated. Consequently, evaluations based solely on independently generated or temporally smoothed demand may overlook operational vulnerabilities that emerge under sudden, context-specific demand concentration.

To address this limitation, Experiment 3 was conducted as a system-level stress test using context-based synchronized demand. The experiment examined system responses under temporally concentrated demand conditions across three scenarios—Festival, Lunch, and Holiday—and three temporal demand conditions—Uniform, Poisson, and Synchronized.

To isolate the effects of these temporal distributions, 900 controlled simulation runs were conducted (three scenarios × three conditions × 100 random seeds). The experimental design employed paired comparisons in which, for each scenario and random seed, the road network, initial vehicle states, and the same realization of routine background demand were held constant. The routine background demand was generated using an NHPP with an average arrival rate of approximately 41 requests per hour [63]. Its temporal and spatial demand patterns were derived from observed station-level boarding and alighting volumes obtained from the Transportation Card Big Data Integrated Information System. The relative proportions of boarding and alighting volumes were used as the origin- and destination-selection probabilities, respectively, thereby incorporating observed public-transit demand patterns into the generation of background DRT requests. Ten target requests were injected into this fixed background environment. Across the paired runs, only the temporal distribution of these ten target requests varied among the Uniform, Poisson, and Synchronized conditions.

System responses were analyzed separately for the target, overall, and background passenger groups. For the target passenger group, delay characteristics were quantified using the mean waiting time, 95th-percentile (P95) waiting time, Gini coefficient [64], and empirical cumulative distribution function (ECDF). At the overall-passenger level, the target group’s share of the total waiting-time burden was analyzed to examine whether the effect of synchronized demand was diluted or asymmetrically represented in aggregate metrics. Finally, the background-passenger analysis examined whether the delay propagated beyond the target group and degraded broader service performance or remained localized. Tables 6–8 summarize the main experimental factors, fixed settings, and evaluation metrics.

**Table 6:** Experimental factors and design levels.

Experimental Factors	
Parameter	Levels
Demand Injection Distribution	Uniform / Poisson / Context-based Synchronized
Spatial Scenario	Festival / Lunch / Holiday

**Table 7:** Fixed parameters for the simulation environment.

Fixed Experimental Setup		
Parameter	Setting	Note
Background Demand	Non-homogeneous Poisson process	Avg. 41 requests/hour
Target Demand	Ten passengers	Injected at t = 1,500 s
Fleet	Six shuttles	Max. nine passengers per shuttle
Simulation Horizon	3,600 s	2,100 s post-injection
Random Seeds	100 per condition	900 runs in total

**Table 8:** Operational state immediately before the target-demand injection at t = 1,500 s.

Operational indicator	Pre-injection state
Cumulative background request entities	17.05 ± 4.10
Cumulative background passengers	46.54 ± 11.35
Active vehicles	3.04 ± 1.50
Idle vehicles	2.96 ± 1.50
Vehicles carrying at least one passenger group	2.06 ± 1.27
Onboard request entities	2.29 ± 1.52
Onboard passengers	6.31 ± 4.36
Onboard passengers per active vehicle	1.92 ± 1.02
Assigned but not yet boarded request entities	1.42 ± 1.23
Assigned but not yet boarded passengers	3.84 ± 3.47
Cumulative rejection rate	0.47% (8/1,705 requests)
Mean elapsed waiting time of waiting request entities	87.66 ± 67.62 s

Values are reported as the mean ± standard deviation across 100 random seeds. The mean elapsed waiting time was calculated using the seeds in which at least one request entity was waiting at the injection time.

Table 8 summarizes the operational state of the system immediately before the target-demand injection at t = 1,500 s. On average, 3.04 of the six vehicles were active and 2.96 were idle. Across the fleet, an average of 6.31 passengers were onboard, while 1.42 request entities, corresponding to 3.84 passengers, had been assigned but had not yet boarded. The cumulative rejection rate before the injection was 0.47% (8 of 1,705 requests). These values suggest that the system was operating under an active background-demand condition without showing signs of full saturation before the ten target requests were introduced. The evaluation metrics and passenger groups used in Experiment 3 are summarized in Table 9.

**Table 9:** Evaluation of metrics and passenger groups.

Evaluation Outputs	
Parameter	Definition
Performance Metrics	Mean wait time / P95 wait time / ECDF / Gini coefficient [64]
Passenger Groups	Target / Background / All passengers

To reflect the geographic characteristics of the target urban and campus transportation networks, three scenarios were defined as follows: Scenario 1 (Festival) represents post-event movement from an event center to a major transit hub; Scenario 2 (Lunch) models daytime peak movement from academic and business areas to a commercial district; and Scenario 3 (Holiday) simulates movement from residential areas to a regional transportation hub. The injection size was fixed at ten one-passenger target request entities for each scenario and random seed. This value was not derived as a general capacity or saturation threshold. Instead, the ten requests were used as a fixed short-term demand perturbation to compare the effects of different temporal arrival patterns while holding the background-demand realization, fleet configuration, and target origins and destinations constant. Accordingly, Experiment 3 evaluates whether the temporal concentration of an identical set of requests produces a localized operational burden, rather than whether ten requests constitute a critical system-wide saturation threshold.

To mitigate the influence of the initial transient state, during which the vehicle positions and background demand distributions were not yet fully stabilized, the target demand was injected 1,500 s into the simulation. Given a total simulation duration of 3,600 s, the injection timing secured a 2,100 s observation window. This timeframe was sufficient to comprehensively analyze the cascading effects of short-term demand concentration on system performance, including passenger waiting times, vehicle repositioning, and cumulative delays.

The temporal distributions of the ten target requests were categorized into two control conditions and one experimental condition. The Uniform distribution (control) dispersed requests at perfectly regular intervals, representing the highest degree of temporal separation. The Poisson distribution (control) modeled the inter-arrival times using an exponential distribution with a mean of 30 s, reflecting a standard probabilistic arrival pattern with irregular intervals. Finally, the context-based Synchronized distribution (experimental) injected all target requests simultaneously at the 1,500 s mark, representing an idealized coordinated demand surge triggered by a specific situational stimulus. The respective cumulative distribution functions are presented in Fig 12 to illustrate the temporal disparities among the three conditions.

**Fig 12:**
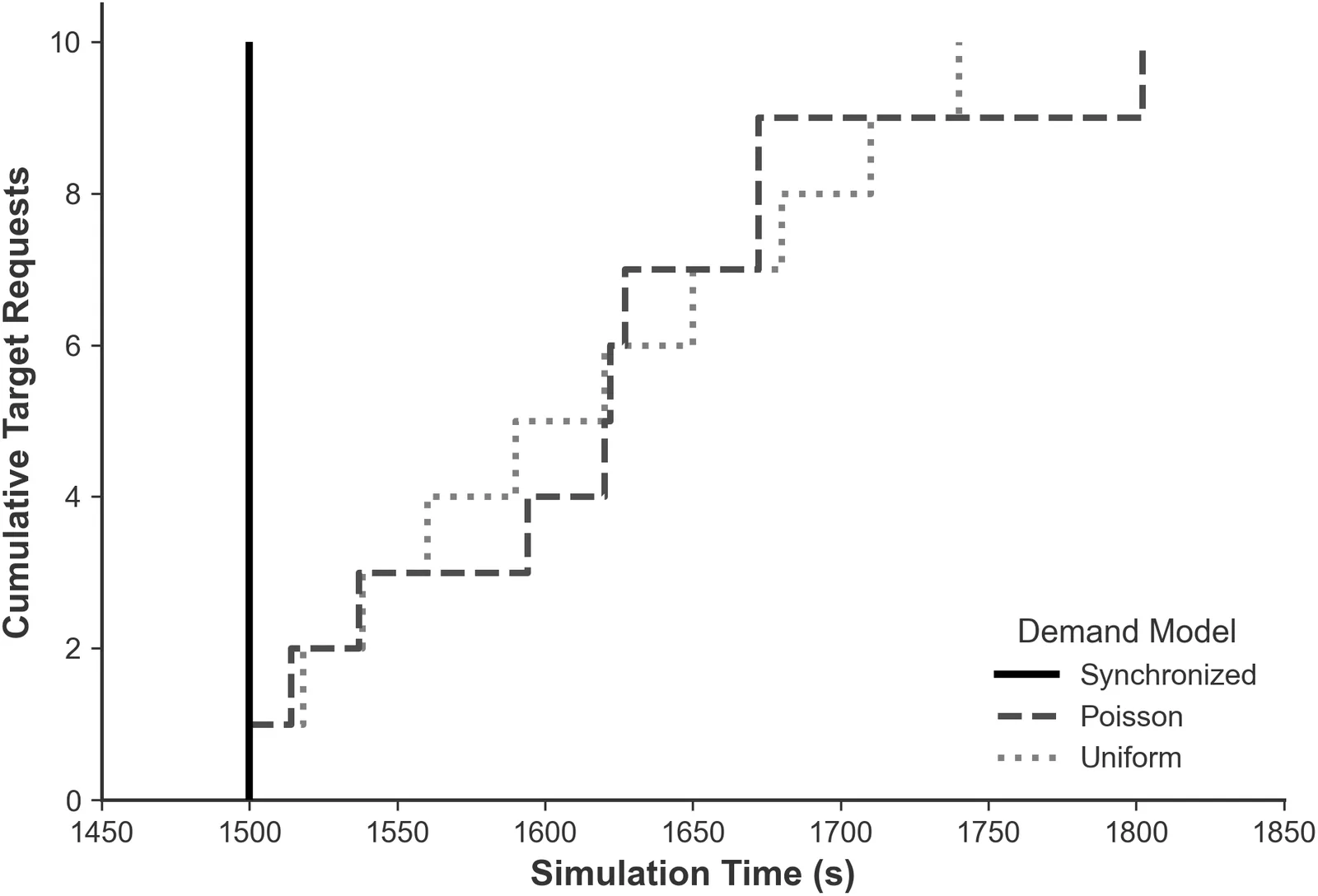
Temporal profiles of the injected target demand under the three experimental conditions.

To identify the localized vulnerabilities induced by demand injections, simulation data were analyzed across three distinct passenger categories. The target passenger group comprised ten specifically injected requests, whereas the background passenger group encompassed routine passengers generated by the baseline NHPP model [63]. Consequently, the overall passenger group represented the aggregate of both target and background passengers. Utilizing this tripartite classification, the subsequent analysis first evaluated the direct impact of context-dependent demand concentration on system performance, beginning with the target passenger group.

Fig 13 presents the mean waiting time of the target passenger group across the three scenarios, utilizing paired comparisons at the random seed level. Across all scenarios, the synchronized demand condition consistently increased the mean waiting time relative to both the Uniform and Poisson conditions. Seed-level paired comparisons revealed a systematic upward shift across most iterations. This pattern suggests that the increase in mean waiting time was not driven solely by a small number of extreme observations but reflected a broader increase across the target passenger group.

**Fig 13:**
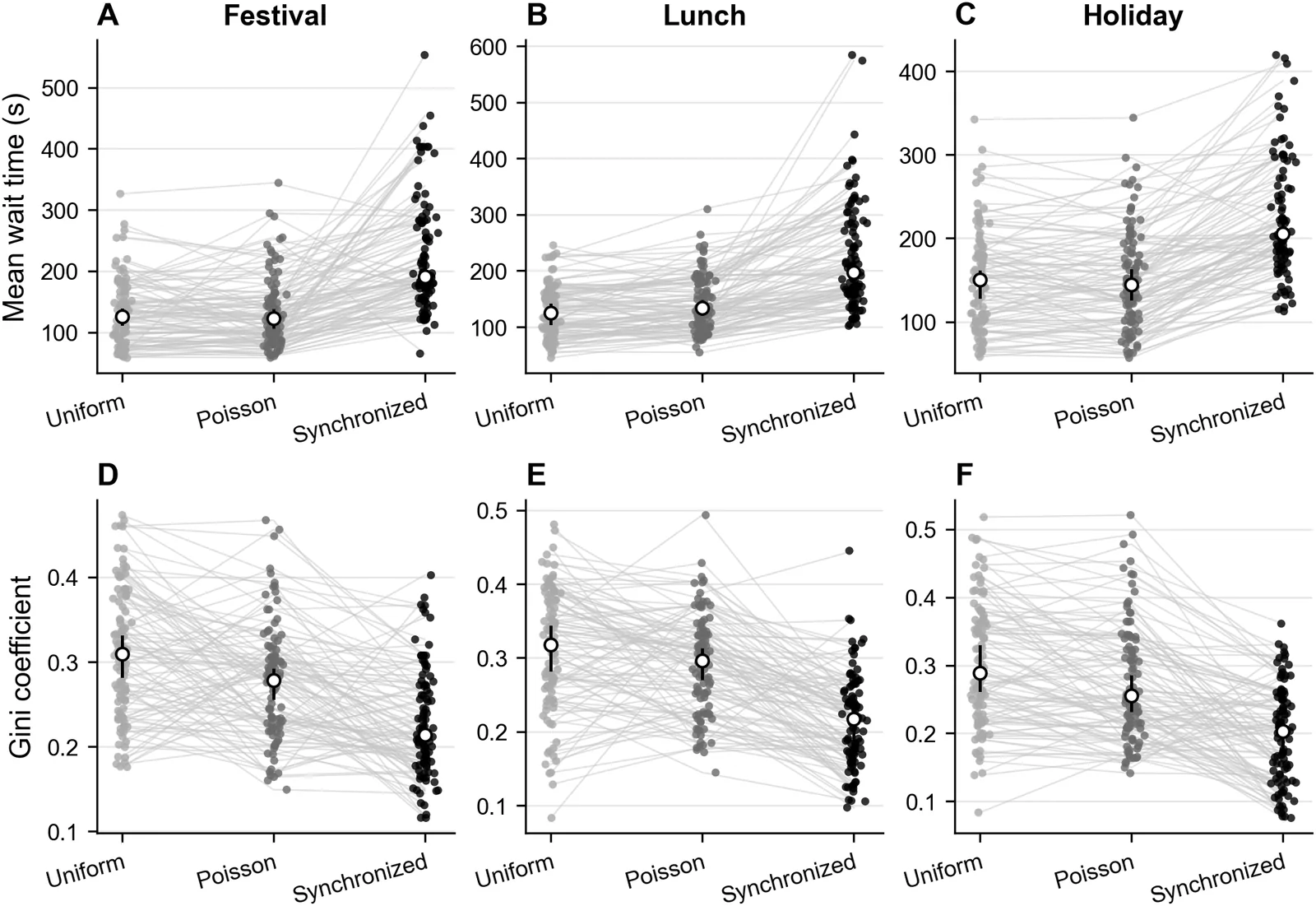
Paired seed-level comparison of target-passenger performance across the three scenarios.

The Gini coefficients shown in Figs 13D–13F were consistently lower under the Synchronized condition than under the Uniform and Poisson conditions. However, a lower Gini coefficient indicates lower relative inequality and does not necessarily imply lower absolute variance or improved service equity [64]. When considered together with the increased mean waiting time and the rightward shift of the ECDF, this pattern suggests that prolonged waiting times were broadly distributed across the target passenger group rather than concentrated in a small subset of passengers.

Fig 14 presents the ECDFs of individual waiting times for the target passenger group, aggregated by scenario. Across all three scenarios, the ECDF curves under the Synchronized condition exhibited a clear rightward shift relative to the Uniform and Poisson conditions. This shift suggests that the increase in waiting time was broadly distributed across the target passenger group rather than being driven solely by a small number of extreme observations. Together, the Gini coefficient and ECDF results indicate a broader distributional shift in target-passenger waiting times under the Synchronized condition.

**Fig 14:**
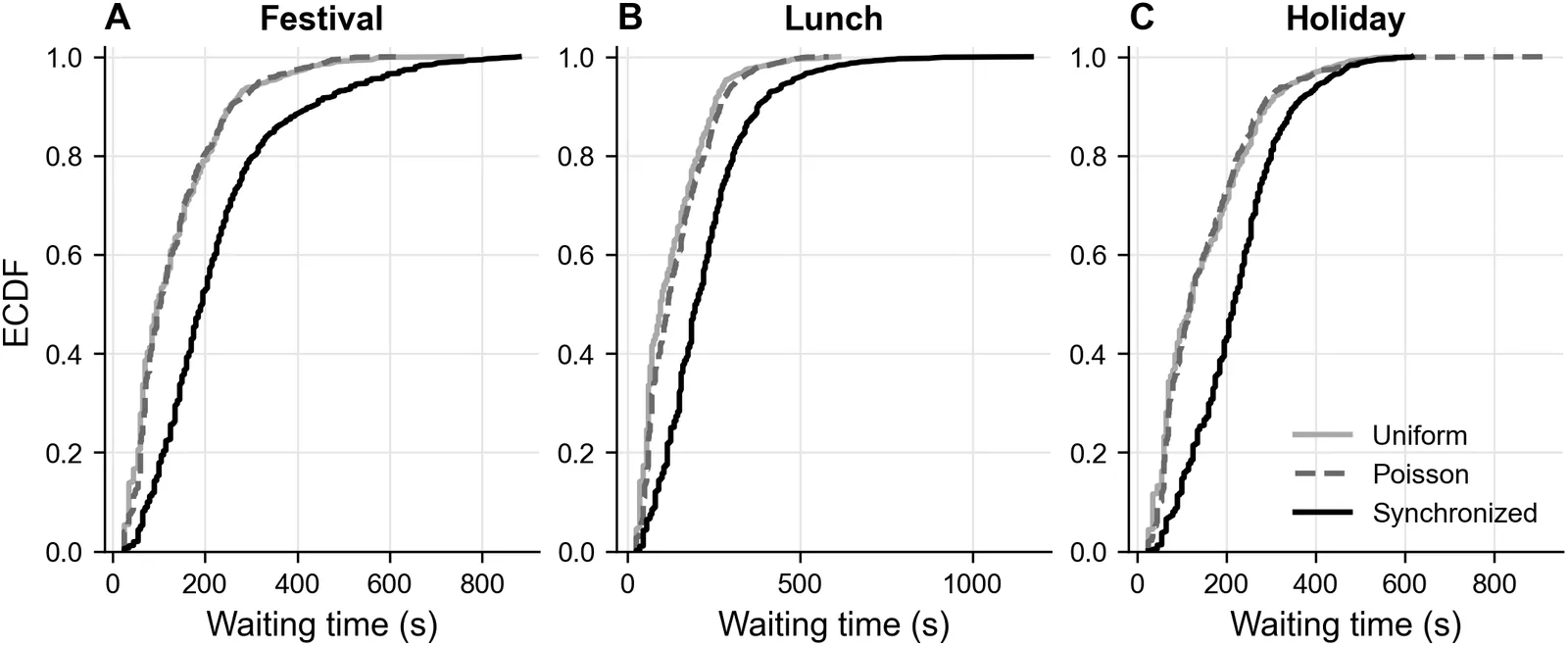
Empirical cumulative distribution functions of waiting times for the target passenger group.

Ultimately, under the tested network, fleet, demand rate, and dispatching constraints, the Synchronized condition increased the mean waiting time while exhibiting lower relative inequality within the target passenger group. Together with the rightward ECDF shift, this suggests that prolonged waiting times were broadly distributed across the target group.

Whereas the preceding analysis focused on the direct effects of synchronized demand on the target passenger group, this subsection examines how these effects were reflected in aggregate service-level metrics. Fig 15 illustrates the changes in the mean and P95 waiting times for the overall passenger group across 100 random seeds.

**Fig 15:**
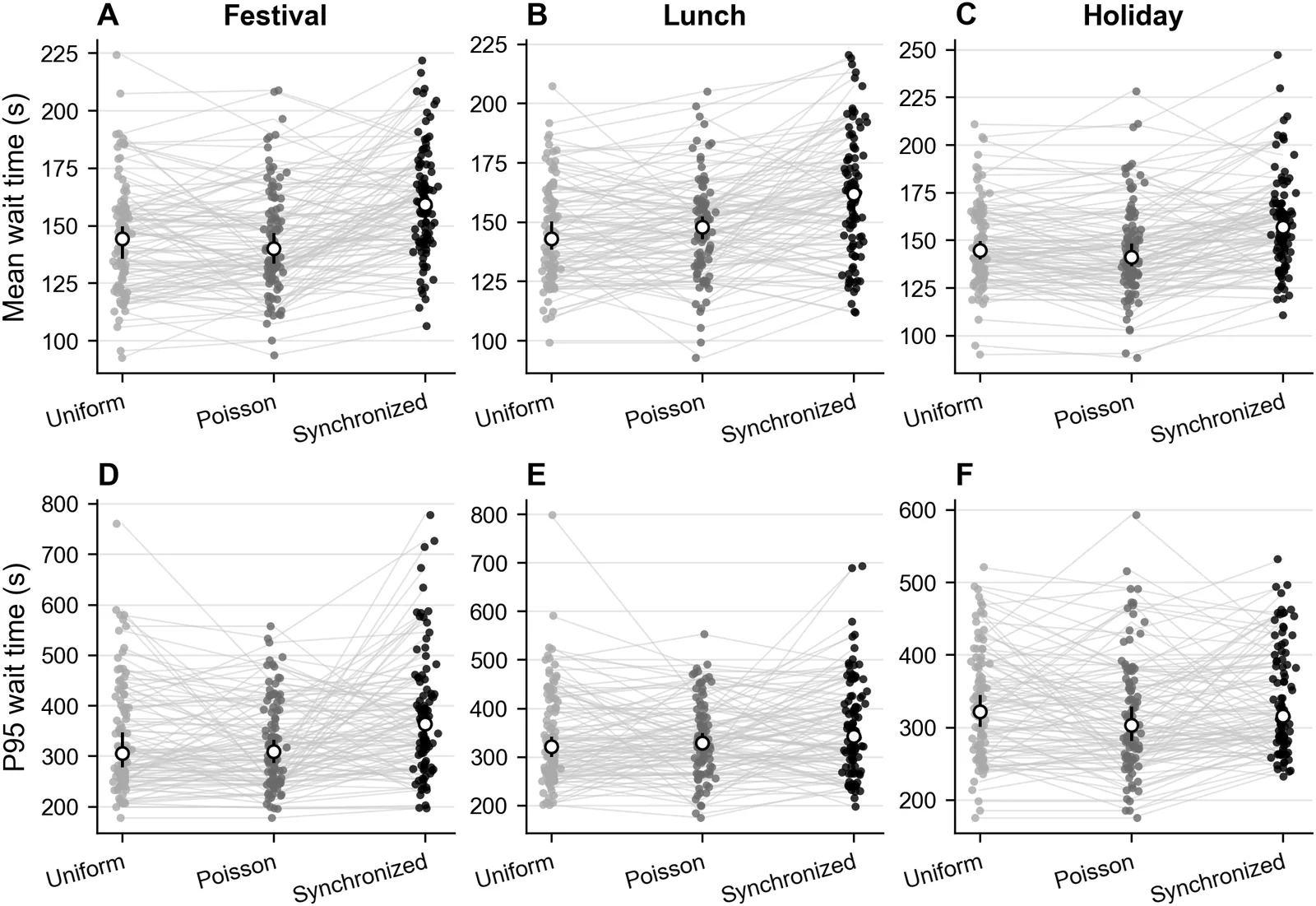
Seed-level variation in system-wide waiting time metrics for all passengers.

To quantify this aggregate-level dilution, the target passenger share and wait burden share were evaluated for each scenario and demand condition. The target passenger share is defined as the proportion of the target passengers relative to the total number of successfully served passengers. Conversely, the burden share represents the proportion of the total passenger waiting time of the system, which is solely attributable to the target group. To ensure statistical robustness against asymmetric distributions and extreme outliers, the median of the seed-level ratios is reported as the representative value for each combination. Furthermore, the paired operational impact was assessed by calculating the seed-level difference between the wait burden share of the Synchronized condition and that of the baseline conditions (Uniform or Poisson), with the median of these differences serving as the final comparative metric.

However, the relative stability of the overall metrics does not imply that the effects of synchronized demand were evenly distributed across passenger groups. Table 10 compares the target group’s share of successfully served passengers with its share of the total waiting time. Although target passengers represented approximately 20% of successfully served passengers, they accounted for a larger share of the total waiting-time burden. The moderate changes observed in aggregate averages therefore reflect the dilution of substantial target-group delays within the larger background-passenger population. This pattern suggests that aggregate-level arithmetic means may underrepresent localized delay burdens and asymmetric delay distributions [64]. To examine whether these effects extended beyond the target group, the following analysis isolates the waiting-time performance of background passengers under the same simulation conditions.

**Table 10:** Target passengers and waiting time burden shares across scenarios and demand conditions.

Scenario	Target share (%)	Uniform (%)	Poisson (%)	Synchronized (%)	Synchronized – Uniform (%)	Synchronized – Poisson (%)
**Festival**	20.4	18.1	18.6	26.5	+8.4	+8.1
**Lunch**	20.8	17.9	19.8	27.2	+9.4	+7.3
**Holiday**	20.4	19.5	22.0	28.5	+8.1	+7.6

Fig 16 illustrates the changes in the mean and P95 waiting times for the background passenger group across 100 random seeds. Background-passenger metrics remained broadly similar across the Uniform, Poisson, and Synchronized conditions, although slight decreases in waiting times were observed for some scenarios and random seeds. Within the tested configuration and evaluated metrics, the operational impact therefore appeared to remain largely localized to the target passenger group rather than propagating into broader background-service degradation. These results suggest that overall average indicators alone may not fully capture localized delay burdens and asymmetric delay distributions.

**Fig 16:**
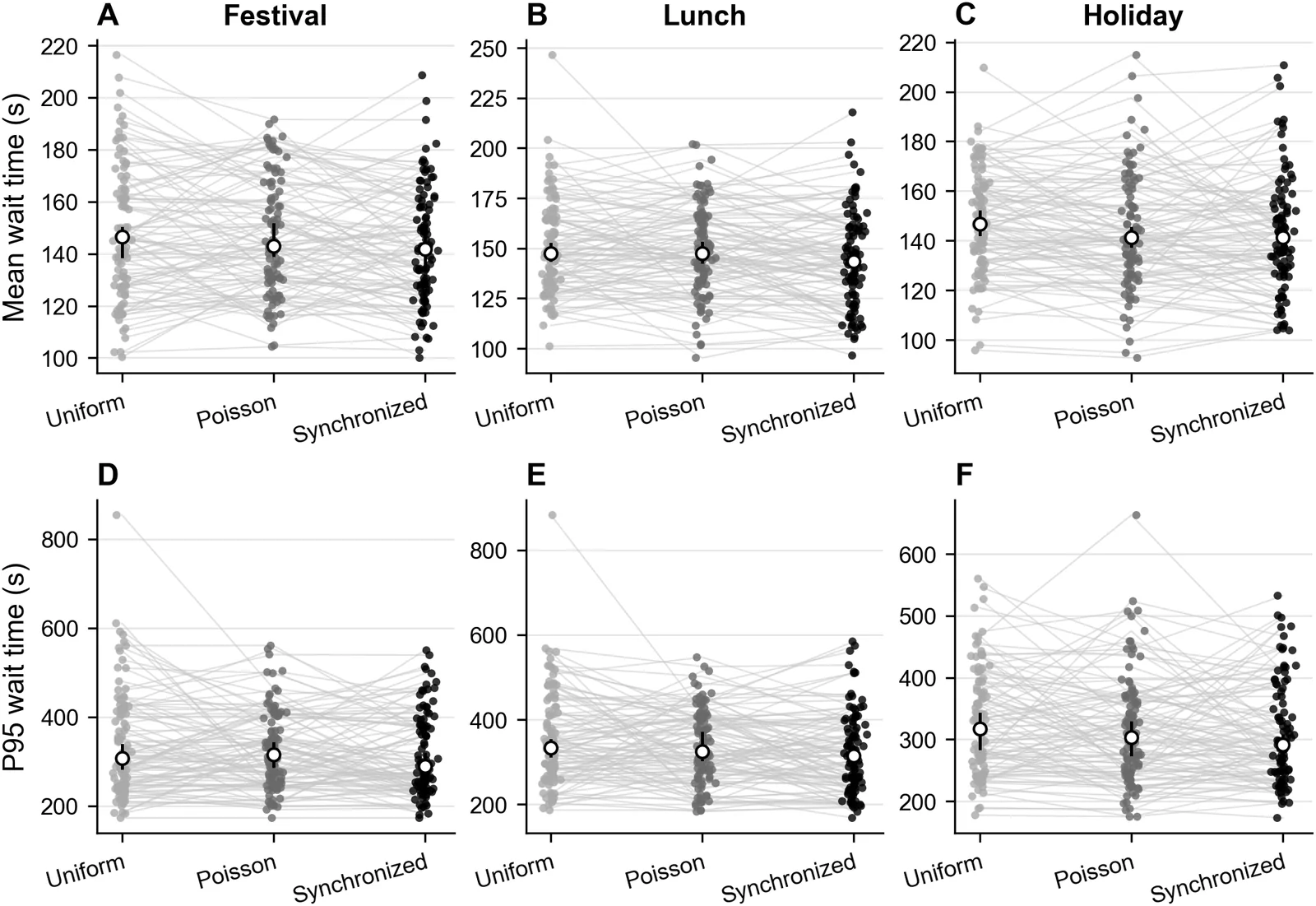
Seed-level variation in waiting time metrics for the background passenger group.

### 4.3 Experimental conclusions

The Synchronized condition consistently increased the mean waiting time of the target passenger group in all three scenarios. This effect was not driven solely by extreme observations but was associated with a broader upward shift in the waiting time distribution. Consequently, the results suggest that context-dependent demand concentration may create localized operational burdens that can be overlooked in evaluations based solely on independently generated demand. Although the aggregate metrics for the overall passenger group exhibited only moderate changes, this apparent stability masked the highly asymmetric delay burden. As listed in Table 10, while target passengers comprised only 20.4–20.8% of the total volume, they accounted for 26.5–28.5% of the total waiting time burden of the system under Synchronized conditions, representing an absolute increase of 7.3–9.4 percentage points relative to the baselines. Furthermore, because the background-passenger metrics remained broadly stable, the operational impact appeared to remain largely localized to the target group rather than propagating system-wide. These results suggest that average-based aggregate metrics alone may not fully capture localized delay burdens or asymmetric delay distributions. Context-based synchronized-demand testing may therefore complement conventional evaluations based on simulation-generated demand.

Experiment 3 should be interpreted as a system-level stress test of the Real-time DRT Simulator rather than as a validation of the LLM-based user and manager interfaces. Its purpose was to examine how a fixed operational configuration responds to alternative temporal distributions of an identical set of target requests.

## 5. Conclusion

This study proposed an LLM-assisted real-time DRT operation framework and conducted stepwise validation across its Service User interface, System Manager interface, and Real-time DRT Simulator layers. The central significance of this architecture lies in its ability to process externally submitted and simulation-generated demands simultaneously within a unified event structure and identical vehicle dispatching and routing logic. Consequently, the real-time simulation environment functions not only as a basic execution tool but also as a validation platform supporting controlled evaluation under operationally relevant conditions. Experiments 1 and 2 evaluated the functional correctness and latency of the LLM-assisted interfaces, whereas Experiment 3 independently evaluated the Real-time DRT Simulator under controlled synchronized-demand conditions. Accordingly, the findings of Experiment 3 are specific to the tested network, fleet, demand rate, and dispatching constraints and do not by themselves establish the general necessity of the overall framework.

Beyond text response generation, the LLM interfaces provide two functional capabilities. For the Service User, the interface converts pre-defined unstructured natural-language inputs, including landmark references, typographical errors, and alternative phrasings, into structured dispatching parameters. For the System Manager, the implemented interface supports pre-defined KPI queries, inter-vehicle comparisons, and domain-knowledge retrieval through natural-language input. However, Experiment 2 evaluated only the function-calling-based KPI and vehicle-status query path; the RAG-based domain-knowledge retrieval path was not separately validated in the present study. The hybrid query interface allows operators to access these pre-defined functions without directly writing SQL queries.

Finally, this study challenges traditional evaluations that rely on smooth probabilistic demand models by executing a system-level stress test using context-based synchronized demand. The real-time simulation-based stress test revealed operational vulnerabilities and asymmetric delay burdens imposed on specific passenger groups, demonstrating the value of context-based synchronized-demand testing for exposing localized risks.

Despite these contributions, some limitations remain. The current functional validation was conducted under controlled scenarios, meaning that scaling the RAG pipeline to handle commercial-level databases or highly concurrent operator requests may introduce unforeseen latency overhead. In addition, empirical studies involving human subjects are required to accurately measure the actual cognitive load reduction provided by these interfaces. Future research will focus on optimizing the computational efficiency of the framework for larger geographic networks and evaluating its long-term stability in fully open real-world operational environments. In relation to the System Manager, the current manager module does not autonomously detect localized bottlenecks or allow operators to modify dispatching policies and feed operational decisions back into the simulator during execution. Future research will therefore extend the framework by incorporating bidirectional operator intervention and closed-loop feedback capabilities.
